# Dissolved Nitrogen Acquisition in the Symbioses of Soft and Hard Corals With Symbiodiniaceae: A Key to Understanding Their Different Nutritional Strategies?

**DOI:** 10.3389/fmicb.2021.657759

**Published:** 2021-06-04

**Authors:** Chloé A. Pupier, Renaud Grover, Maoz Fine, Cécile Rottier, Jeroen A. J. M. van de Water, Christine Ferrier-Pagès

**Affiliations:** ^1^Marine Department, Centre Scientifique de Monaco, Monaco, Monaco; ^2^Collège Doctoral, Sorbonne Université, Paris, France; ^3^The Goodman Faculty of Life Sciences, Bar-Ilan University, Ramat Gan, Israel; ^4^The Interuniversity Institute for Marine Science in Eilat, Eilat, Israel

**Keywords:** nitrogen, nutrition, octocoral, mesophotic, stable isotopes

## Abstract

Nitrogen is one of the limiting nutrients for coral growth and primary productivity. Therefore, the capacity of different associations between corals and their algal symbionts (Symbiodiniaceae) to efficiently exploit the available nitrogen sources will influence their distribution and abundance. Recent studies have advanced our understanding of nitrogen assimilation in reef-building scleractinian (hard) coral-Symbiodiniaceae symbioses. However, the nutrient metabolism of other coral taxa, such as Alcyoniina (soft corals), remains underexplored. Using stable isotope labeling, we investigated the assimilation of dissolved nitrogen (i.e., ammonium, nitrate, and free amino acids) by multiple species of soft and hard corals sampled in the Gulf of Aqaba in shallow (8–10 m) and mesophotic (40–50 m) reefs. Our results show that dissolved nitrogen assimilation rates per tissue biomass were up to 10-fold higher in hard than in soft coral symbioses for all sources of nitrogen. Although such differences in assimilation rates could be linked to the Symbiodiniaceae density, Symbiodiniaceae species, or the C:N ratio of the host and algal symbiont fractions, none of these parameters were different between the two coral taxa. Instead, the lower assimilation rates in soft coral symbioses might be explained by their different nutritional strategy: whereas soft corals may obtain most of their nitrogen via the capture of planktonic prey by the coral host (heterotrophic feeding), hard corals may rely more on dissolved nitrogen assimilation by their algal symbionts to fulfill their needs. This study highlights different nutritional strategies in soft and hard coral symbioses. A higher reliance on heterotrophy may help soft corals to grow in reefs with higher turbidity, which have a high concentration of particles in suspension in seawater. Further, soft corals may benefit from lower dissolved nitrogen assimilation rates in areas with low water quality.

## Introduction

Nitrogen (N) is essential for the life and growth of all organisms on Earth, as it is required for the biosynthesis of key cellular components. However, N is a growth-limiting nutrient in oligotrophic marine ecosystems, such as coral reefs (de Goeij et al., 2013). Corals are the primary reef-building and habitat-forming species in these marine environments and require a steady supply of nutrients for growth and reproduction. As a consequence, they have evolved as meta-organisms or holobionts (Rohwer et al., 2002; Bosch and McFall-Ngai, 2011), in which the host is associated with an assemblage of microorganisms. These microbial communities are notably involved in the protection against pathogens and in the efficient uptake and recycling of the few available nutrients (Rädecker et al., 2015).

The coral animal itself is mainly capable of assimilating particulate and dissolved organic N [e.g., dissolved free amino acids (DFAA) and urea] (Grover et al., 2006, 2008). However, microbes involved in all steps of the N cycle have been found in the microbiota of corals. For example, N-fixing microorganisms (Benavides et al., 2017) convert N_2_ gas into bioavailable ammonium (NH_4_^+^), and numerous microbes, including the common hard and soft coral symbiont *Endozoicomonas*, can perform dissimilatory nitrate (NO_3_^–^) reduction into NH_4_^+^ (Neave et al., 2014). Finally, the algal symbionts belonging to the family Symbiodiniaceae (LaJeunesse et al., 2018) are the main assimilation site of the dissolved inorganic N forms (DIN), such as NH_4_^+^ and NO_3_^–^ (Muscatine et al., 1979; Grover et al., 2002, 2003; Pernice et al., 2012).

The assimilation of dissolved N (DN) by corals is, however, influenced by both the environmental conditions and the algal symbionts they are associated with. For example, some genera of Symbiodiniaceae take up nutrients more efficiently than others (Baker A.C. et al., 2013; Leal et al., 2015; Pernice et al., 2015), and high light levels promote their DN assimilation (Grover et al., 2008). In contrast, elevated seawater temperatures reduce DIN availability in surface waters due to enhanced water column stratification, which prevents the upwelling of nutrients recycled in deep waters (Behrenfeld et al., 2006). It also induces coral bleaching and thereby impairs the ability of the remaining symbionts to take up DIN (Godinot et al., 2011; Krueger et al., 2018).

As N assimilation is positively correlated with primary productivity, the capacity of different coral-dinoflagellate symbioses to efficiently exploit the different N sources available will influence their distribution and abundance in a given environment. Therefore, a thorough understanding of the acquisition and allocation of N within different associations and different environments [e.g., shallow (high light) versus mesophotic (low light) reefs] is essential to improve our prediction of the ecological conditions under which each association is favored. However, the nutritional ecology of coral-dinoflagellate symbioses, particularly regarding the N metabolism, has mostly focused on a few main reef-building scleractinian (hard) coral species (Muscatine et al., 1989; Hoegh-Guldberg and Williamson, 1999; Mills et al., 2004; Baker D.M. et al., 2013; Ezzat et al., 2017). On the other hand, other coral taxa such as soft corals (sub-order Alcyoniina) have been largely overlooked (Schlichter, 1982; Burris, 1983), despite being the second most common benthic group on many reefs and therefore recognized as key taxa (Schubert et al., 2017). The lack of data on soft corals, along with the use of different data normalization metrics in studies on soft and hard corals, limit our ability to predict how nutrient conditions may favor the growth of one group over the other. High abundances of octocorals (soft corals or gorgonians) have, however, been observed on eutrophicated reefs, where high concentrations of nutrients in the water do not favor the growth of hard corals (Bell, 1992; Gast et al., 1999; Baum et al., 2016; Vollstedt et al., 2020). This dominance of soft corals in disturbed reef ecosystems suggests that they may have a different nutrient metabolism or nutritional relationship with their symbionts compared with hard corals. Also, corals tend to increase their reliance on heterotrophy with depth (mesophotic reefs), although this might be species-dependent (Muscatine and Kaplan, 1994).

To gain better insights into the extent to which soft corals rely on their symbionts for their nutrition, we investigated how these symbiotic associations assimilate different DN forms, and how this may be impacted by depth and temperature. We hypothesized (1) that Symbiodiniaceae in symbiosis with soft corals can use all DN forms, and (2) that low irradiance (as measured at mesophotic depths) or elevated temperatures will negatively affect the assimilation rates of DN, as previously observed for hard corals. In addition, we conducted a comparative study with hard corals, allowing a better understanding of the differences in the functional and nutritional ecology of soft and hard corals, with the underlying hypothesis that soft corals rely less on the assimilation of DN than hard corals because they are generally considered as more heterotrophic. A higher reliance on heterotrophy may help soft corals to grow in reefs with high turbidity, and they may benefit from low DN assimilation rates in areas with low water quality.

## Materials and Methods

### Biological Material

The study was conducted at the Inter-University Institute (IUI) for Marine Sciences (Eilat, Israel) in November 2018. The experiment was performed with three species of hard corals (*Galaxea fascicularis*, *Stylophora pistillata*, and *Seriatopora hystrix*) and three species of soft corals (*Litophyton arboreum*, *Rhytisma fulvum fulvum*, and *Sarcophyton* sp.). Coral fragments (5 cm height) were collected by SCUBA diving on the shallow (8–10 m depth) and mesophotic (40–50 m depth) reefs adjacent to the IUI. Temperature and nutrient levels were not different between the shallow and mesophotic reefs at the time of collection. Temperature ranged from 24.7 to 25.4°C during the experiment. Nitrate concentrations ranged from 0.17 to 0.25 μM, whereas dissolved inorganic phosphorus and ammonium were below 0.2 μM (data obtained from the National Monitoring Program of the Gulf of Eilat^1^.

**FIGURE 1:**
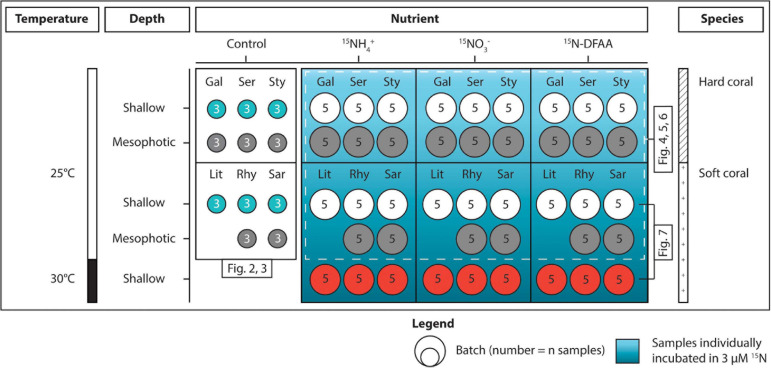
Overview of the experimental design. Corals sampled at shallow and mesophotic depths were distributed across three conditions (Control, incubated in ^15^N for 5 h, and maintained at 30°C for 1 week × incubated in ^15^N for 5 h). The seawater used for the incubations was enriched with 3 μM of either ^15^NH_4_^+^, ^15^NO_3_^–^, or ^15^N-DFAA. The first three species [*Galaxea fascicularis* (Gal), *Seriatopora hystrix* (Ser), *Stylophora pistillata* (Sty)] are hard corals and the last three [*Litophyton arboreum* (Lit), *Rhytisma fulvum fulvum* (Rhy), *Sarcophyton* sp. (Sar)] are soft corals. Colors of the batches match with those displayed on the figures. DFAA = dissolved free amino acids.

Eighteen nubbins per coral species were collected from different colonies at both shallow and mesophotic depths (except for *L. arboreum*, which was only found in shallow waters) (Figure 1). Three nubbins were immediately frozen for the determination of Symbiodiniaceae genus and density, chlorophyll concentration, C and N content, and natural isotopic signatures. The remaining fragments were maintained in 11 outdoor aquaria (one aquarium per species and depth) at the Red Sea Simulator facility (described in Bellworthy and Fine, 2018) for a maximum of 2 days. Aquaria were continuously supplied with seawater directly pumped from the reef. Corals were maintained under the natural light cycle at light levels corresponding to their *in situ* depth (ca. 450 and 30 μmole photons m^–2^ s^–1^ at midday, for shallow and mesophotic environments respectively), by adjusting light levels with shade cloth.

In addition to the conditions described above, three additional aquaria were prepared 2 weeks in advance, each containing five nubbins per species of soft corals collected from the shallow reef (5 m). Aquaria were maintained under the exact same conditions as above, except that temperature was gradually increased (+0.5°C per day) up to 30°C (+5°C than the control condition). Corals were kept at this higher temperature for 1 week prior to the incubations with ^15^N tracers, run at the same time as the other incubations.

### Determination of the Genus of Symbiodiniaceae Symbionts

The genus of the algal symbionts hosted by the corals was investigated following the protocol of Santos et al. (2002) with some modifications. In short, genomic DNA was extracted from coral samples using the DNeasy Plant mini kit (Qiagen, Germany). Domain V of the 23S rRNA gene of the chloroplast was PCR amplified using the primers 23S1M13 (5′-CACGACGTTGTAAAACGACGGCTGTAACTATAACGGTCC-3′) and 23S2M13 (5′-GGATAACAATTTCACACAGGC CATCGTATTGAACCCAGC-3′) (Santos et al., 2002). PCRs were performed in 50 μL volumes containing 1× PCR Buffer (10 mM Tris–HCl of pH 8.3 and 50 mM KCl), 1.5 mM MgCl_2_, 0.2 mM dNTPs, 0.2 μM of each primer, 2 U Platinum *Taq* polymerase (Invitrogen, Thermo Fischer Scientific, United States), and 25 ng of template DNA. Reactions were performed in a ProFlex PCR System (Applied Biosystems, United States) under the following conditions (initial denaturing period of 2 min at 95°C, 40 cycles consisting of 95°C for 45 s, 55°C for 45 s, and 72°C for 1 min, and a final extension period of 7 min at 72°C). Amplicons were purified using MinElute PCR Purification kit (Qiagen, Germany) and were sent to Eurofins Genomics (Germany) for Sanger sequencing using the 23S1M13 forward primer. Sequences obtained were trimmed to remove (1) the bases matching the 23S2M13 reverse primer sequence (up to 39 bp) and (2) the first eight nucleotides because of the larger number of ambiguous base calls (N). BLASTn (Altschul et al., 1990) was used (*megablast* algorithm, default parameters) against the non-redundant NCBI sequence database [limited to the Symbiodiniaceae (taxid: 252141)] to identify the genus of the main Symbiodiniaceae symbiont. This marker has been commonly used for assessments of Symbiodiniaceae diversity (e.g., Bayliss et al., 2019).

### Incubations

^15^N stable isotope tracers were used to assess the assimilation rates of dissolved inorganic and organic N sources by hard and soft corals, as per Grover et al. (2002; 2003; 2008) and Ezzat et al. (2017). To this end, 15 L of 0.45 μm-filtered seawater (FSW) were enriched with either ^15^NH_4_Cl, ^15^NO_3_Na, or ^15^N-DFAA (all at 98 atom %^15^N, Sigma-Aldrich, United States) to reach a final N concentration of 3 μM. Five 250 mL beakers were prepared per species, depth, and N compound and filled with 200 mL of ^15^N-enriched seawater. One coral fragment was added to each beaker. Incubations were performed at the Red Sea simulator facility under the natural irradiance of shallow and mesophotic depths. Corals were incubated at 25°C for 5 h, between 11:00 am and 04:00 pm to cover the maximal daily irradiance. Similarly, an incubation was also carried out at 30°C on the additional set of shallow soft corals that were pre-incubated at 30°C (as described above), to assess the effects of temperature on the assimilation rates of N by soft corals. At the end of the incubations, coral nubbins were rinsed in 0.2 μm-FSW and frozen at −80°C until further analysis.

### Sample Processing

Soft coral samples were processed as described in Pupier et al. (2018). Briefly, they were freeze-dried, weighed, and crushed into powder. For hard corals, the tissue was removed from the skeleton using an airbrush and FSW and homogenized with a Potter-Elvehjem tissue grinder, and subsequently freeze-dried, weighed, and crushed into powder. The skeleton was kept for the determination of the skeletal surface area using the wax dipping technique, which provides an accurate approximation of the tissue surface area of a coral nubbin, with the benefits of being both fast and cheap (Veal et al., 2010). For both hard and soft coral samples, a fraction of the powder (∼30 mg) was used for the determination of the ash-free dry weight (AFDW), as described below, and the remaining tissue was homogenized in 10 mL distilled water with a Potter-Elvehjem tissue grinder. The host and algal symbiont fractions of each sample were separated through a series of centrifugations (Grover et al., 2003). The algal symbiont pellet was resuspended in 10 mL distilled water. For the control samples, a 500 μL subsample was taken for the determination of algal symbiont density using a improved Neubauer hemocytometer (Marienfeld Superior, Germany) and light microscope. In addition, a 2.5 mL subsample from the algal symbiont fraction was used to determine the chlorophyll concentration according to Jeffrey and Humphrey (1975), using a spectrophotometer (SAFAS, Monaco). The remaining fractions of the host and algal symbionts were subsequently freeze-dried and weighed. Approximately 500 μg of lyophilized host and algal symbiont material were transferred to tin caps for analysis of the ^15^N enrichment, carbon (C), and N content and the natural δ^15^N signatures using an Integra II isotope ratio mass spectrometer (Sercon, United Kingdom). The total amount of N assimilated in each compartment was then determined using the modified equations of Dugdale and Wilkerson (1986) according to Grover et al. (2002). These equations compare the N enrichment obtained in the incubations with ^15^N with the natural isotopic values of control corals, sampled at the beginning of the experiment. N (either derived from NH_4_^+^ or NO_3_^–^) translocation percentages (T_*S*_) were calculated. The calculations were based on the assumptions that (1) Symbiodiniaceae are the primary site of fixation for NH_4_^+^ and NO_3_^–^ (Grover et al., 2002, 2003; Pernice et al., 2012), and the transfer of organic N compounds (N-NH_4_^+^ or N-NO_3_^–^) occurs from the algal symbionts to the host fraction (which comprises the host and all the other associated microorganisms), and that (2) the loss of N is negligible. In addition, a correction was applied for N-NH_4_^+^ since the host fraction can directly assimilate up to 4% of the total NH_4_^+^ assimilated by the holobiont (Pernice et al., 2012; Tang et al., 2020). T_*S*_ were expressed in % and were calculated as:

$${\text{T}}_{S\,\left(\text{N}-{\text{NH}}_{4}^{+}\right)}=\frac{\rho_{\text{H}}-\left(\left(\rho_{S}+\rho_{\text{H}}\right)\times 0.04\right)}{\left(\rho_{S}+\rho_{\text{H}}\right)}\times 100$$

$${\text{T}}_{S\,\left(\text{N}-{\text{NO}}_{3}^{-}\right)}=\frac{\rho_{\text{H}}}{\left(\rho_{S}+\rho_{\text{H}}\right)}\times 100$$

where ρ_*S*_ is the N assimilation rate of the algal symbionts and ρ_*H*_ the N assimilation rate of the host fraction.

All data were normalized to the AFDW of the nubbins, which was used as a proxy for biomass. The subsample of lyophilized material was first weighed to obtain the dry weight, and then combusted at 450°C for 4 h in a Thermolyne 62700 muffle furnace (Thermo Fisher Scientific, United States). AFDW was determined as the difference between the dry weight and ash weight of the subsample and extrapolated to the total weight of the nubbin. AFDW was used for normalization because it was found to be the most reliable metric for the normalization of physiological parameters of soft corals (Pupier et al., 2018). Moreover, since N is assimilated into coral biomass, it is therefore more relevant to estimate the assimilation rates per biomass. This is in agreement with the observations of Edmunds and Gates (2002), who also concluded that normalizing data to biomass is most effective in accounting for coral size in comparative studies.

### Statistical Analyses

Analyses were performed using the R environment for statistical computing and graphics (R Core Team, 2017). Generalized linear models were used to test (1) the effect of species, depth, and N source on the assimilation rates of N by the host or the algal fractions at 25°C, (2) the effect of species, temperature, and N source on the assimilation rates of N by the host or algal fractions in shallow soft corals, and (3) the effect of species and depth on tissue descriptors in control corals, as well as on translocation percentages. Generalized linear models (family = Gaussian) were fitted on data transformed using the Box-Cox transformation procedure (Supplementary Table 1) as implemented in the R-package *MASS* (Ripley et al., 2013). Compliance with the assumptions of a normal distribution of the model residuals and homoscedasticity was verified using, respectively, the Shapiro–Wilk test and Levene’s test as implemented in the R-package *car* (Fox and Weisberg, 2018). The *emmeans* package (Lenth et al., 2020) was used to calculate estimated marginal means and compute biologically relevant contrasts. The Benjamini-Hochberg procedure (Benjamini and Hochberg, 1995) was used for multiplicity adjustments of *p*-values.

## Results

Detailed outcomes of the statistical analyses are reported in Supplementary Table 2 for the tissue descriptors and Supplementary Table 3 for the assimilation rates.

Analyses of the chloroplast 23SrRNA gene sequences of the Symbiodiniaceae revealed that colonies of *L. arboreum* and *S. pistillata* collected from shallow reefs harbored *Symbiodinium* sp. (>98.94% identity similarity, *E* value = 0.0, GenBank accession number KF740693.1), whereas colonies of *S. pistillata* from the mesophotic zone, as well as all other coral species regardless of depth, were associated with *Cladocopium* sp. (>99.31% identity similarity, *E* value = 0.0, GenBank accession number MK696599.1).

**FIGURE 2:**
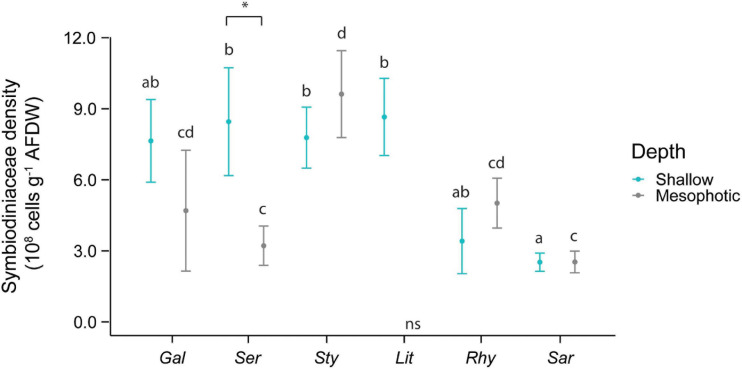
Symbiodiniaceae density in the species investigated. The first three species [*Galaxea fascicularis* (Gal), *Seriatopora hystrix* (Ser), *Stylophora pistillata* (Sty)] are hard corals and the last three [*Litophyton arboreum* (Lit), *Rhytisma fulvum fulvum* (Rhy), *Sarcophyton* sp. (Sar)] are soft corals. Significant differences between depths are displayed with an asterisk (^∗^ for *p* < 0.05). Significant differences between species are distinguished with letters by depth. AFDW = ash-free dry weight. ns = not sampled.

An interactive effect of species and depth was observed for the densities of algal symbiont cells per tissue biomass (Figure 2). On shallow reefs, *Sarcophyton* sp. harbored significantly lower levels of Symbiodiniaceae than *L. arboreum*, *S. hystrix*, and *S. pistillata*. Densities in mesophotic corals were significantly higher in *S. pistillata* than *Sarcophyton* sp. and *S. hystrix*. In addition, algal symbiont densities in *S. hystrix* were significantly lower at mesophotic than at shallow depth.

Total chlorophyll concentration per tissue biomass was overall similar in all the different coral species at mesophotic depth. At shallow depth, however, algal symbionts of *S. hystrix* contained higher levels of chlorophyll than those associated with all soft coral species (Supplementary Figure 1A). Symbionts of mesophotic colonies of *S. hystrix* also had a higher chlorophyll concentration per total tissue biomass than those of shallow colonies (Supplementary Figure 1A).

Chlorophyll concentrations per algal symbiont cell showed significant variation across depths and species. At shallow depth, symbionts associated with *R. f. fulvum* and *Sarcophyton* sp. contained higher levels of chlorophyll than those associated with the different hard coral species or with *L. arboreum* (Supplementary Figure 1B). Contrastingly, algal symbiont cells of *S. pistillata* contained significantly lower amounts of chlorophyll in comparison with symbionts associated with all other coral species at mesophotic depths. Algal symbionts of *G. fascicularis* and *S. hystrix* contained significantly higher concentrations in chlorophyll per symbiont cell at the mesophotic depth than at shallow depth (Supplementary Figure 1B).

**FIGURE 3:**
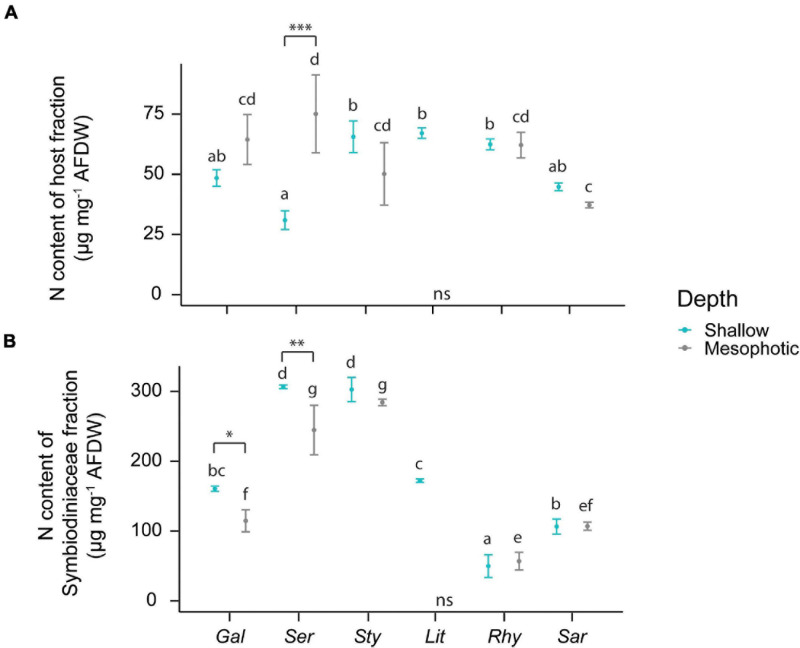
Elemental composition of the host tissue and Symbiodiniaceae fraction in the species investigated. **(A)** N content of the host tissue. **(B)** N content of the Symbiodiniaceae fraction. The first three species [*Galaxea fascicularis* (Gal), *Seriatopora hystrix* (Ser), *Stylophora pistillata* (Sty)] are hard corals and the last three [*Litophyton arboreum* (Lit), *Rhytisma fulvum fulvum* (Rhy), *Sarcophyton* sp. (Sar)] are soft corals. Significant differences between depths are displayed with asterisks (^∗^ for *p* < 0.05; ^∗∗^ for *p* < 0.01; ^∗∗∗^ for *p* < 0.001). Significant differences between species are distinguished with letters by depth. AFDW = ash-free dry weight. ns = not sampled.

The N content of the host fraction was lower in *S. hystrix* than in *S. pistillata*, *L. arboreum*, and *R. f. fulvum* collected from the shallow reef (Figure 3A). At mesophotic depths, however, *S. hystrix* contained more N in its host fraction than *Sarcophyton* sp. In the algal symbionts, the N content varied depending on coral host species and depths. Overall, the N contents of the Symbiodiniaceae were lower in *G. fascicularis*, *R. f. fulvum*, and *Sarcophyton* sp. compared with *S. hystrix* and *S. pistillata* at both shallow and mesophotic depths (Figure 3B).

Differences in N and C content in both the coral and Symbiodiniaceae fractions (Figure 3 and Supplementary Figures 2C,D, respectively) explained the differences in the C:N ratios between species (Supplementary Figure 2). Overall, tissues of hard corals and *L. arboreum* had C:N ratios of ∼5, which was significantly lower than *Sarcophyton* sp., (∼7) and *R. f. fulvum* (∼16) (Supplementary Figure 2A). Similar patterns were observed in the C:N ratios of the algal symbionts. In general, algal symbionts of all hard corals and *Sarcophyton* sp. had C:N ratios varying between 6 and 10, which was higher than the C:N ratio of *L. arboreum* symbionts (∼5) but lower than the C:N ratios of *R. f. fulvum* symbionts (∼15–20). The higher C:N ratios observed in *R. f. fulvum* were likely due to a relatively high C content in the host fraction, in contrast to a relatively low N content in the Symbiodiniaceae fraction.

**FIGURE 4:**
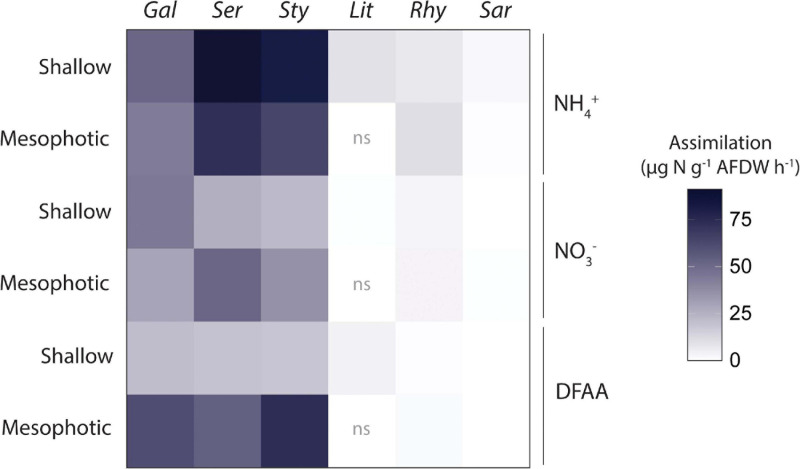
Total N assimilation rates by the holobionts investigated. Data displayed are means of the assimilation by the host and the assimilation by the Symbiodiniaceae fraction added together. From left to right: hard corals (Gal = *Galaxea fascicularis*, Ser = *Seriatopora hystrix*, Sty = *Stylophora pistillata*) and soft corals (Lit = *Litophyton arboreum*, Rhy = *Rhytisma fulvum fulvum*, Sar = *Sarcophyton* sp.). ns = not sampled. AFDW = ash-free dry weight. DFAA = Dissolved free amino acids.

**FIGURE 5:**
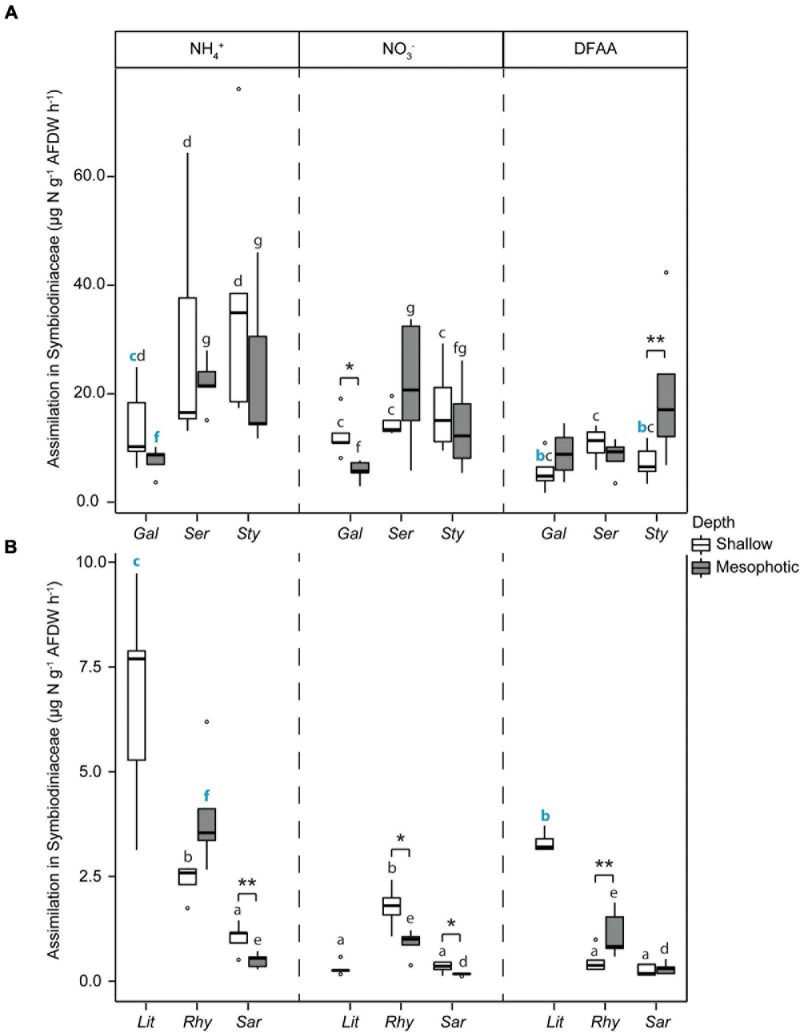
Assimilation rates of dissolved N in the Symbiodiniaceae fraction in **(A)** hard corals [*Galaxea fascicularis* (Gal), *Seriatopora hystrix* (Ser), *Stylophora pistillata* (Sty)] and **(B)** soft corals [*Litophyton arboreum* (Lit), *Rhytisma fulvum fulvum* (Rhy), *Sarcophyton* sp. (Sar)]. Significant differences between depths are displayed with asterisks (* for *p* < 0.05; ** for *p* < 0.01). For each N source taken independently, significant differences between species are distinguished with letters by depth. Blue bold letters highlight a similar rate between hard and soft coral species. AFDW = ash-free dry weight. ns = not sampled. DFAA = dissolved free amino acids. ns = not sampled.

Total N assimilation rates (host + symbionts) were ca. 10-fold higher in hard than in soft corals for all investigated N sources (Figure 4). Assimilation rates were overall higher in algal symbionts of hard compared to soft corals with the following exceptions (Figure 5): algal symbionts of shallow colonies of *L. arboreum* and mesophotic colonies of *R. f. fulvum* had similar NH_4_^+^ assimilation rates than algal symbionts of *G. fascicularis* at the corresponding depths. DFAA assimilation rates were not different between the algal symbionts of shallow colonies of *L. arboreum*, *S. pistillata*, and *G. fascicularis*. Algal symbionts of *Sarcophyton* sp. had lower assimilation rates of NH_4_^+^ and NO_3_^–^ at mesophotic than at shallow depth. A similar pattern was observed for NO_3_^–^ assimilation by the algal symbionts of *R. f. fulvum*. However, assimilation rates of DFAA were higher at mesophotic than at shallow depth in *R. f. fulvum*.

Translocation percentages of N (either derived from NH_4_^+^ or NO_3_^–^) assimilated by the algal symbionts and transferred as organic N compounds (N-NH_4_^+^ or N-NO_3_^–^) to the host fraction (which comprises the host and all the other associated microorganisms) were calculated (Table 1). Differences were observed between depths only for NO_3_^–^ in soft corals and there was no differences were found between hard and soft coral holobionts (Supplementary Table 3).

**TABLE 1:** Translocation percentages (T_*S*_) of organic nitrogen compounds derived from the assimilation of dissolved inorganic nitrogen (either NH_4_^+^ or NO_3_^–^) by the algal symbionts in hard (Gal = *Galaxea fascicularis*, Ser = *Seriatopora hystrix*, Sty = *Stylophora pistillata*) and soft coral (Lit = *Litophyton arboreum*, Rhy = *Rhytisma fulvum fulvum*, Sar = *Sarcophyton* sp.) holobionts from shallow and mesophotic reefs.

	**T${}_{\text{S}}\,\left(\text{N}-{\text{NH}}_{4}^{+}\right)$(%)**			**T${}_{\text{S}}(\text{N}-{\text{NO}}_{3}^{-}$)(%)**		
	**Shallow**	**Mesophotic**	**30°C**	**Shallow**	**Mesophotic**	**30°C**
Gal	70 ± 5	79 ± 1	–	72 ± 1	80 ± 2	–
Ser	63 ± 5	66 ± 2	–	45 ± 6	57 ± 9	–
Sty	52 ± 6	61 ± 4	–	42 ± 12	62 ± 2	–
Lit	29 ± 9	–	48 ± 11	49 ± 4	–	56 ± 1
Rhy	61 ± 4	58 ± 5	57 ± 7	52 ± 8	77 ± 3	43 ± 7
Sar	49 ± 2	61 ± 4	40 ± 3	53 ± 4	73 ± 2	45 ± 2

*Calculations are based on the assumptions that (1) Symbiodiniaceae are the primary site of assimilation and the transfer of organic N compounds (N-NH_4_^+^ or N-NO_3_^–^) occurs from the algal symbionts to the host fraction (which comprises the host and all the other associated microorganism), and that (2) the loss of N is negligible. In addition, a correction was applied for NH_4_^+^ since the host fraction can directly assimilate up to 4%. N-NH_4_^+^ = N derived from the assimilation of ammonium. N-NO_3_^–^ = N derived from the assimilation of nitrate. – = not sampled.*

**FIGURE 6:**
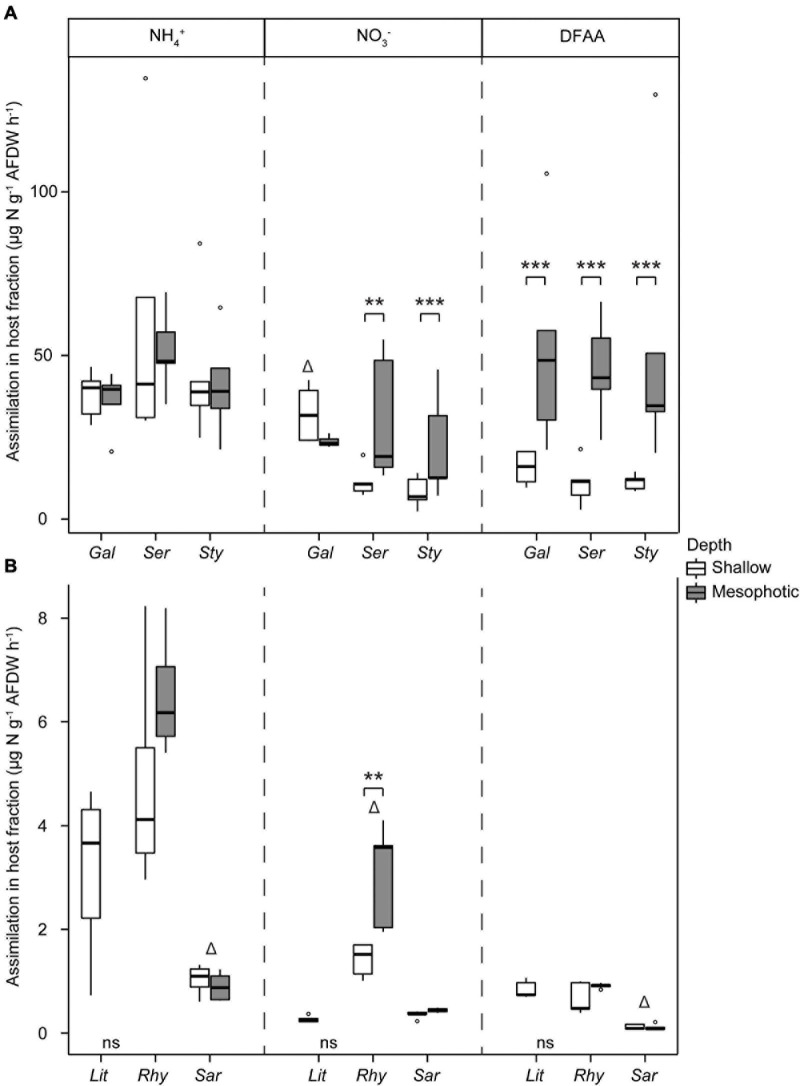
Assimilation rates of dissolved N in the host tissue in **(A)** hard corals [*Galaxea fascicularis* (Gal), *Seriatopora hystrix* (Ser), *Stylophora pistillata* (Sty)] and **(B)** soft corals [*Litophyton arboreum* (Lit), *Rhytisma fulvum fulvum* (Rhy), *Sarcophyton* sp. (Sar)]. Significant differences between depths are displayed with asterisks (** for *p* < 0.01; *** for *p* < 0.001). Δ shows the species that differentiates from the other two within its group (i.e., either hard or soft corals, for a single N source). AFDW = ash-free dry weight. DFAA = dissolved free amino acids. ns = not sampled.

The assimilation of N per tissue biomass in the host fraction was ca. 10-fold higher in hard than in soft corals for all investigated N sources (Figure 6). At each depth, hard coral hosts had similar assimilation rates of N-NH_4_^+^, N-NO_3_^–^, and DFAA, except *G. fascicularis*, which had higher N-NO_3_^–^ assimilation compared with the other species at shallow depth (Supplementary Table 3 and Figure 6A). Rates of DFAA assimilation into the host fraction were consistently higher at mesophotic than at shallow depth (Figure 6A and Supplementary Table 3). A similar effect of depth was observed on the rates of N-NO_3_^–^ assimilation into the host fraction of *S. hystrix* and *S. pistillata*. No effect of depth was observed for N-NH_4_^+^ assimilation rates. Concerning the soft coral species, N-NH_4_^+^ and DFAA assimilation rates into the host fraction were significantly lower in *Sarcophyton* sp. than in the two other species, while N-NO_3_^–^ assimilation rates were higher in *R. f. fulvum* (Figure 6B and Supplementary Table 3). There was no difference in the assimilation rates of N-NH_4_^+^, N-NO_3_^–^, and DFAA between shallow and mesophotic soft coral host fractions, except for N-NO_3_^–^ assimilation rates in *R. f. fulvum*, which were higher in colonies from the mesophotic zone (Supplementary Table 3).

**FIGURE 7:**
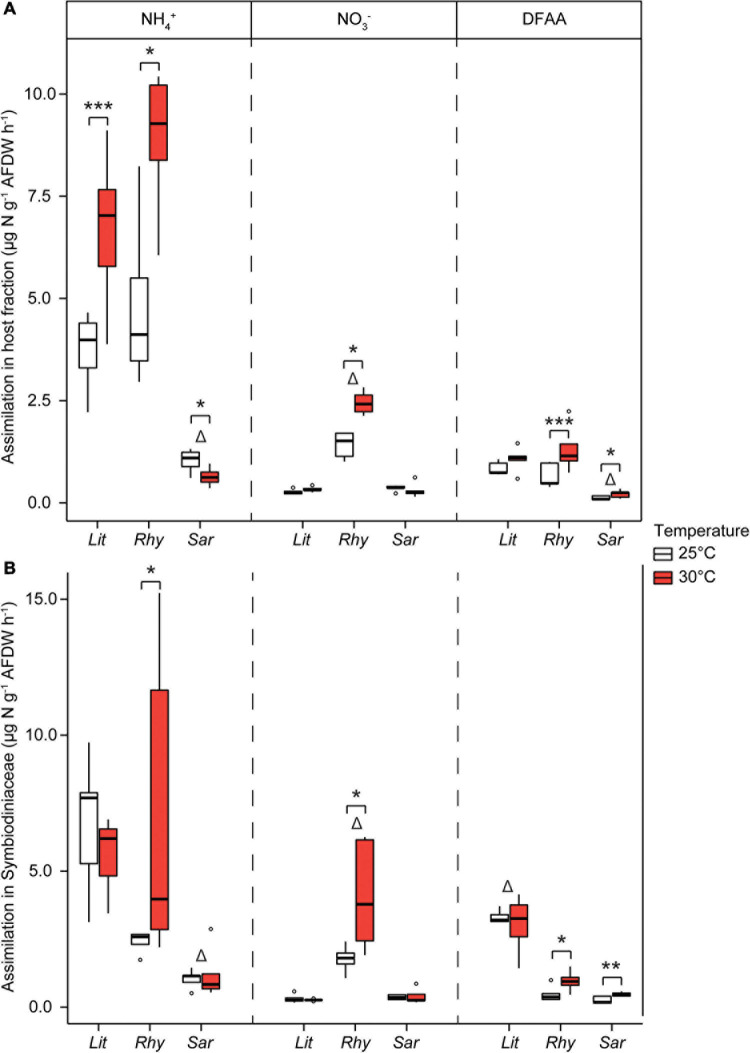
Assimilation rates of dissolved N in shallow soft corals exposed at two temperatures, in **(A)** the host tissue and **(B)** the Symbiodiniaceae fraction [*Litophyton arboreum* (Lit), *Rhytisma fulvum fulvum* (Rhy), *Sarcophyton* sp. (Sar)]. Significant differences between temperatures are displayed with asterisks (* for *p* < 0.05; ** for *p* < 0.01; *** for *p* < 0.001). Δ shows the species that differentiates from the other two within its group (i.e., either host or Symbiodiniaceae fraction, for a single N source). AFDW = ash-free dry weight. DFAA = dissolved free amino acids. ns = not sampled.

To investigate the N assimilation at the maximal summer temperature in the Gulf of Aqaba (30°C), soft corals collected from the shallow reefs were exposed to 30°C for 1 week and compared with the corals at ambient/winter (25°C) temperatures. Assimilation rates of NH_4_^+^ into the algal symbiont and host fractions of *R. f. fulvum* as well as in the host fraction of *L. arboreum* were significantly higher under increased seawater temperatures (Figure 7). Assimilation rates of NO_3_^–^ were enhanced in the algal symbiont and host fractions of *R. f. fulvum*, while assimilation rates of DFAA were higher in both the algal symbionts and host fraction of *R. f. fulvum* and *Sarcophyton* sp. at 30°C. At elevated temperatures, the differences in N assimilation between species were consistent with the findings at 25°C. Namely, *Sarcophyton* sp. assimilated NH_4_^+^ (both algal symbiont and host fractions) and DFAA (host fraction) at lower rates than the other species, and the assimilation rates of NO_3_^–^ were significantly higher in *R. f. fulvum* (both fractions), while the assimilation of DFAA was higher in the symbiont fraction of *L. arboreum*.

## Discussion

Nitrogen availability is a major factor determining the growth, productivity, and overall fitness of coral holobionts (Rädecker et al., 2015). Within these holobionts, the assimilation of inorganic N dissolved in seawater (NH_4_^+^ and NO_3_^–^) is mainly performed by their algal symbionts, as they are the most active site for NH_4_^+^ fixation (Grover et al., 2002; Pernice et al., 2012), and are the primary site of fixation for NO_3_^–^, because animals do not possess NO_3_^–^ reductases (Miller and Yellowlees, 1989). Following assimilation, the algal symbionts transfer N to their coral host in the form of organic N compounds (Rädecker et al., 2015). The assimilatory capacity of the endosymbionts depends, however, on the transition of N from seawater through the host tissue to the symbiont cells. In contrast, dissolved organic N forms, such as amino acids (DFAA), can be directly assimilated by both the coral host and algal symbionts into proteins (Grover et al., 2008). Therefore, both partners play a role in N assimilation, which together determines the capacity of the holobiont to take up nutrients. This study highlights a different capacity of soft and hard corals to assimilate dissolved N forms. The lower assimilation rates per unit biomass measured in soft coral holobionts are likely due to different nutritional strategies and morphological traits, such as a lower reliance on the algal symbionts for N acquisition. In addition, we observed an increased assimilation of N with increasing depth or seawater temperature for most but not all coral-Symbiodiniaceae associations.

### Differences in N Assimilation Between Soft and Hard Coral-Symbiodiniaceae Symbioses

Our study highlights 10-fold lower rates of dissolved N assimilation per unit biomass in soft coral holobionts than in hard coral holobionts, whose uptake rates are comparable to previous studies (Supplementary Figure 3; Grover et al., 2002; Pernice et al., 2012; Ezzat et al., 2017). Uptake rates of dissolved nutrients have previously been linked to the Symbiodiniaceae densities or Symbiodiniaceae species, as some are less efficient than others in nutrient acquisition (Baker A.C. et al., 2013; Ezzat et al., 2017). In our study, however, the genus and density of Symbiodiniaceae were similar between the soft and hard corals investigated and could thus not explain the differences observed in N assimilation rates. Percentages of translocation of organic N compounds derived from the assimilation of NH_4_^+^ and NO_3_^–^ by the algal symbionts were also not significantly different between the hard and soft coral groups. In addition, the lower assimilation rates of dissolved N (DFAA) in soft corals suggest that they have lower uptake capacities or, alternatively, lower needs for these N sources than hard corals. However, the similar N contents per unit biomass in soft and hard coral tissue and Symbiodiniaceae do not support different N needs in one of the symbiotic associations, although the tissue turnover can be different between the two coral groups.

Several morphological and metabolic characteristics can contribute to the higher N assimilation rates in hard coral compared to soft coral symbioses. At the host level, soft corals are primarily characterized by the absence of a calcium carbonate skeleton and only possess sclerites as calcified structures (Fabricius and Alderslade, 2001). In hard corals, the deposition of a skeleton (calcification) generates protons that have to be neutralized to avoid tissue acidification (Comeau et al., 2013). Crossland and Barnes (1974) and Biscéré et al. (2018) suggested that ammonia may be involved in proton neutralization, through the ornithine cycle and urea production, therefore possibly promoting the uptake of DIN by the symbionts. In addition, soft corals comprise a large coenenchyme mostly constituted of mesoglea, which can represent a significant barrier to transepithelial diffusion of molecules (oxygen in Bradfield and Chapman, 1983; bicarbonate in Furla et al., 1998). The expansion state, together with the low surface area to body volume ratio, usually found for soft corals exhibiting fleshy and massive morphologies, are also not in favor of exchanges through the epidermal tissue (Kirschner, 1991; Shick, 1991; Fabricius and Klumpp, 1995). At the symbiont level, Symbiodiniaceae associated with hard corals living in shallow waters generally exhibit higher rates of photosynthesis than those in symbiosis with soft corals (e.g., Fabricius and Klumpp, 1995; Pupier et al., 2019b). As N uptake is proportional to the algal symbiont’s photosynthetic activity (e.g., Grover et al., 2002), the higher photosynthesis levels of hard coral symbionts (Pupier et al., 2019b) can explain their higher rates of N assimilation.

Besides Symbiodiniaceae, other eukaryotic as well as prokaryotic microbes may also be involved in the cycling of N within the coral holobiont. For example, “new” N can be introduced via N_2_ fixation activity by symbiotic diazotrophic bacteria, but this has so far not been observed in soft corals (Pupier et al., 2019a). Soft corals may also harbor a higher abundance of microbes involved in the dissimilatory nitrate reduction to ammonium (DNRA) process, recycling N and in turn providing DIN to their host (D’Elia and Wiebe, 1990). Higher levels of N recycling via DNRA may limit the need for additional “new” N uptake. N may also be lost via denitrification processes, which may alleviate excess N availability in coral holobionts (Tilstra et al., 2019). The microbial role in N assimilation and recycling, both in soft and hard corals, remains to be further investigated.

Although microbes may play a role in the N cycling within the coral holobiont, our observations suggest that the contribution of the microbial symbionts to N assimilation is rather limited in soft corals. Soft corals thus likely rely primarily on heterotrophic feeding on particulate organic matter to meet their N requirements. Soft corals, and octocorals in general, are indeed known to capture high amounts of phyto- and zooplankton as well as other forms of particulate organic matter suspended in the surrounding seawater (Sebens and Koehl, 1984; Fabricius et al., 1995a, b, 1998; Fabricius and Dommisse, 2000; Migné and Davoult, 2002; Piccinetti et al., 2016). To test the contribution of autotrophy and heterotrophy to the N demand in soft and hard corals, tracers can be applied, such as Compound Specific Isotope Analysis of the amino acids (Ferrier-Pagès et al., 2021).

### Effect of Depth and Seawater Temperature on N Assimilation Rates

Symbiodiniaceae associated with soft and hard corals showed the same trends with depth, regardless of their host species. They either maintained or increased their chlorophyll content (per unit biomass or per symbiont cell) at mesophotic depth, which is likely a strategy to increase the capture of light energy (Kahng et al., 2019) for acclimation to the lower light levels (Mass et al., 2007; Ziegler et al., 2015). Also, their translocation of organic N compounds derived from the assimilation of NO_3_^–^ was enhanced at mesophotic depth in three out of the six coral species. This finding is in contrast with previous observations that light stimulates N uptake and assimilation (Grover et al., 2002). Such a discrepancy may be linked to the normalization used (i.e., the surface area in previous studies and the tissue biomass in this study). Finally, the assimilation of DFAA was higher in the host fraction of the hard coral species and in the Symbiodiniaceae of *R. f. fulvum* at mesophotic depth. Such higher assimilation of organic N corresponds with a reduced reliance on autotrophy due to reducing light levels with increasing depth in mesophotic corals. Consequently, they may rely more on heterotrophy (Williams et al., 2018) while hard corals may also obtain more N from diazotrophic bacteria (Bednarz et al., 2018). Overall, the enhanced assimilation rates of N at mesophotic depth suggest higher N needs in mesophotic colonies to sustain their metabolism, but this hypothesis remains to be further investigated.

Increased temperatures tended to enhance the assimilation of N in both the host and algal symbiont fractions of soft corals, which is in agreement with a previous study performed on the N assimilation rates in *S. pistillata* at high temperatures (29°C) (Godinot et al., 2011). Such increases in N assimilation can be of a mechanistic nature, for example a thermal optimum reached for the enzymes involved in N assimilation. However, it could also be linked to an increased metabolism at high temperature (Gillooly et al., 2001), although this hypothesis remains to be tested.

### Ecological Implications

Overall, this study corroborates that soft coral-Symbiodiniaceae associations depend on another N source than dissolved nitrogenous compounds to meet their N requirements. This suggests that soft corals rely primarily on heterotrophy. This may be especially true for species bearing polyps with low surface area to volume ratios because they have lower photosynthetic rates, and thus rely less on autotrophy, than species bearing polyps with high surface area to volume ratios (Baker et al., 2015; Rossi et al., 2018). A lower reliance on their algal symbionts may help soft corals to grow on reefs with higher turbidity or sedimentation regimes, as these conditions decrease the amount of light received by corals (decreased autotrophy) but present high concentrations of suspended particles in the seawater (McClanahan and Obura, 1997). Further, the lower DN assimilation rates by soft coral-Symbiodiniaceae associations may favor their survival in areas with low water quality. For example, the algal symbionts may shift from a N limited to a phosphorus-starved state under excess N availability (Wiedenmann et al., 2012), resulting in phospholipids in the chloroplast’s thylakoid membranes being substituted by sulfolipids. Since phospholipids are essential to the stability of the membranes under heat-stress conditions (Tchernov et al., 2004), increased N availability may therefore increase the bleaching susceptibility of corals (Wiedenmann et al., 2012). In addition, excess N can reduce photosynthate translocation rates by Symbiodiniaceae (Ezzat et al., 2015), leading to the starvation of the coral host. As assimilation rates of N are relatively low in soft corals, it is however unlikely that excess N would enter the tissues and disrupt the symbiosis. This may be a reason why soft corals are more abundant than hard corals on reefs exposed to high concentrations of dissolved inorganic nutrients (Baum et al., 2016). Further studies investigating the N and phosphorus budget of soft and hard coral holobionts along a eutrophication gradient are needed to investigate how nutrients may explain the ecological niches of these two coral groups.

## Data Availability Statement

The original contributions presented in the study are included in the article/Supplementary Material, further inquiries can be directed to the corresponding author/s.

## Author Contributions

CP, RG, and CF-P designed the study. CP, MF, RG, and CF-P performed the experiment. CP, CR, and RG performed the sample analyses. CP and JW analyzed the data. CF-P and MF acquired funding. All authors wrote the manuscript.

## Conflict of Interest

The authors declare that the research was conducted in the absence of any commercial or financial relationships that could be construed as a potential conflict of interest.

## Footnotes

1 https://iui-eilat.huji.ac.il/Research/NMPMeteoData.aspx
