# Supplementary material for: Dissolved Nitrogen Acquisition in the Symbioses of Soft and Hard Corals With Symbiodiniaceae: A Key to Understanding Their Different Nutritional Strategies?
Source: Front Microbiol. 2021 Jun 4;12:657759. doi: 10.3389/fmicb.2021.657759 (PMC8211778; doi:10.3389/fmicb.2021.657759)
Supplement: Supplementary file 1 [file Data_Sheet_1.pdf]

Supplementary material

Dissolved nitrogen acquisition in the symbioses of soft and hard corals with Symbiodiniaceae: a key to understanding their different nutritional strategies?

Pupier CA, Grover R, Fine M, Rottier C, van de Water JAJM, Ferrier-Pagès C

Table S1: Lambda of Box-Cox transformation used for statistical tests.

Variable	Figure	Lambda
Symbiodiniaceae density	2	0.060606
N content of the host fraction	3A	0.141414
N content of the algal symbionts	3B	0.828282
DN assimilation rate in the host (per AFDW)	5	-0.060606
DN assimilation rate in the symbionts (per AFDW)	6	-0.060606
DN assimilation rate in the host (per AFDW)	7A	-0.020202
DN assimilation rate in the symbionts (per AFDW)	7B	-0.060606
Chlorophyll concentration (per AFDW)	S1A	0.343434
Chlorophyll concentration (per symbiont cell)	S1B	0.424242
C:N of the host fraction	3A	-0.383838
C:N of the algal symbiont fraction	3A	-1.191919
DN assimilation rate in the host (per surface)	S2A	0.383838
DN assimilation rate in the symbionts (per surface)	S2B	0.141414

Table S2: Statistical analysis for the tissue descriptors. *Galaxea fascicularis* (Gal), *Seriatopora hystrix* (Ser), *Stylophora pistillata* (Sty) are hard corals and *Litophyton arboreum* (Lit), *Rhytisma fulvum fulvum* (Rhy), *Sarcophyton* sp. (Sar) are soft corals. Significant differences are displayed in bold. AFDW = ash-free dry weight. DFAA = dissolved free amino acids.

	Symbiodiniaceae density (Figure 1)					
**GLM**			**Df**	**LR Chisq**	**Pr(>Chi)**	
		Depth	1	1.297	0.2547	
		Species	5	24.085	**0.0002**	
		Depth:Species	4	9.927	**0.0417**	
**Emmeans**		**Pairwise comparison**	**estimate**	**SE**	**z.ratio**	**p.value**
	Gal	Shallow - Mesophotic	-2.338	1.3	-1.793	0.0730
	Rhy		1.617	1.3	1.240	0.2151
	Sar		-0.037	1.3	-0.029	0.9773
	Ser		-3.247	1.3	-2.489	**0.0128**

	Sty		0.684	1.3	0.524	0.6000
	Shallow	Gal - Lit	-0.492	1.3	-0.377	0.9133
		Gal - Rhy	2.985	1.3	2.289	**0.0414**
		Gal - Sar	3.576	1.3	2.742	**0.0229**
		Gal - Ser	-0.312	1.3	-0.239	0.9133
		Gal - Sty	-0.170	1.3	-0.131	0.9133
		Lit - Rhy	3.477	1.3	2.666	**0.0230**
		Lit - Sar	4.068	1.3	3.119	**0.0204**
		Lit - Ser	0.179	1.3	0.138	0.9133
		Lit - Sty	0.321	1.3	0.246	0.9133
		Rhy - Sar	0.591	1.3	0.453	0.9133
		Rhy - Ser	-3.297	1.3	-2.528	**0.0287**
		Rhy - Sty	-3.155	1.3	-2.419	**0.0333**
		Sar - Ser	-3.888	1.3	-2.981	**0.0204**
		Sar - Sty	-3.746	1.3	-2.872	**0.0204**
		Ser - Sty	0.142	1.3	0.109	0.9133
	Mesophotic	Gal - Rhy	-0.970	1.3	-0.744	0.8569
		Gal - Sar	1.275	1.3	0.977	0.7036
		Gal - Ser	0.596	1.3	0.457	0.9713
		Gal - Sty	-3.192	1.3	-2.448	0.0719
		Rhy - Sar	2.245	1.3	1.721	0.2651
		Rhy - Ser	1.566	1.3	1.201	0.5745
		Rhy - Sty	-2.222	1.3	-1.704	0.2651
		Sar - Ser	-0.679	1.3	-0.520	0.9713
		Sar - Sty	-4.467	1.3	-3.425	**0.0092**
		Ser - Sty	-3.789	1.3	-2.905	**0.0276**
	**Chlorophyll concentration per AFDW (Figure S1A)**					
**GLM**			**Df**	**LR Chisq**	**Pr(>Chi)**	
		Depth	1	9.2506	**0.0024**	
		Species	5	24.8715	**0.0001**	
		Depth:Species	4	7.7589	0.1008	
**Emmeans**		**Pairwise comparison**	**estimate**	**SE**	**z.ratio**	**p.value**
	Gal	Shallow - Mesophotic	-4.37	4.86	-0.899	0.3686
	Rhy		-5.69	4.86	-1.169	0.2424
	Sar		1.62	4.86	0.333	0.7393
	Ser		-17.05	4.86	-3.506	**0.0005**
	Sty		-7.59	4.86	-1.56	0.1189
	Shallow	Gal - Lit	-8.47	4.86	-1.74	0.1363
		Gal - Rhy	-11.54	4.86	-2.373	0.0529
		Gal - Sar	-8.99	4.86	-1.847	0.1213
		Gal - Ser	7.55	4.86	1.551	0.1812
		Gal - Sty	1.93	4.86	0.398	0.7402
		Lit - Rhy	-3.08	4.86	-0.633	0.6586
		Lit - Sar	-0.52	4.86	-0.107	0.9148
		Lit - Ser	16.01	4.86	3.292	**0.0050**
		Lit - Sty	10.4	4.86	2.138	0.0697

		Rhy - Sar	2.56	4.86	0.526	0.6912
		Rhy - Ser	19.09	4.86	3.925	**0.0013**
		Rhy - Sty	13.48	4.86	2.771	**0.0210**
		Sar - Ser	16.53	4.86	3.399	**0.0050**
		Sar - Sty	10.92	4.86	2.245	0.0619
		Ser - Sty	-5.61	4.86	-1.154	0.3390
	Mesophotic	Gal - Rhy	-12.86	4.86	-2.643	0.1232
		Gal - Sar	-2.99	4.86	-0.615	1.0000
		Gal - Ser	-5.13	4.86	-1.055	0.8740
		Gal - Sty	-1.28	4.86	-0.263	1.0000
		Rhy - Sar	9.86	4.86	2.028	0.2130
		Rhy - Ser	7.72	4.86	1.588	0.4212
		Rhy - Sty	11.58	4.86	2.38	0.1297
		Sar - Ser	-2.14	4.86	-0.44	1.0000
		Sar - Sty	1.72	4.86	0.353	1.0000
		Ser - Sty	3.86	4.86	0.793	1.0000
	**Chlorophyll concentration per symbiont cell (Figure S1B)**					
**GLM**			**Df**	**LR Chisq**	**Pr(>Chi)**	
		Depth	1	30.252	**<.0001**	
		Species	5	56.285	**<.0001**	
		Depth:Species	4	40.793	**<.0001**	
**Emmeans**		**Pairwise comparison**	**estimate**	**SE**	**z.ratio**	**p.value**
	Gal	Shallow - Mesophotic	-0.007737	0.00162	-4.762	**<.0001**
	Rhy		0.000572	0.00162	0.352	0.725
	Sar		0.000874	0.00162	0.538	0.5904
	Ser		-0.010898	0.00162	-6.708	**<.0001**
	Sty		-0.002792	0.00162	-1.718	0.0857
	Shallow	Gal - Lit	-2.01E-03	0.00162	-1.238	0.2698
		Gal - Rhy	-8.11E-03	0.00162	-4.989	**<.0001**
		Gal - Sar	-8.01E-03	0.00162	-4.931	**<.0001**
		Gal - Ser	2.14E-03	0.00162	1.317	0.2562
		Gal - Sty	7.42E-04	0.00162	0.457	0.6941
		Lit - Rhy	-6.09E-03	0.00162	-3.751	**0.0004**
		Lit - Sar	-6.00E-03	0.00162	-3.693	**0.0004**
		Lit - Ser	4.15E-03	0.00162	2.555	**0.0177**
		Lit - Sty	2.75E-03	0.00162	1.695	0.1352
		Rhy - Sar	9.42E-05	0.00162	0.058	0.9538
		Rhy - Ser	1.02E-02	0.00162	6.306	**<.0001**
		Rhy - Sty	8.85E-03	0.00162	5.446	**<.0001**
		Sar - Ser	1.02E-02	0.00162	6.248	**<.0001**
		Sar - Sty	8.75E-03	0.00162	5.388	**<.0001**
		Ser - Sty	-1.40E-03	0.00162	-0.86	0.4497
	Mesophotic	Gal - Rhy	2.03E-04	0.00162	0.125	1
		Gal - Sar	6.00E-04	0.00162	0.37	1
		Gal - Ser	-1.02E-03	0.00162	-0.629	1
		Gal - Sty	5.69E-03	0.00162	3.501	**0.0035**

		Rhy - Sar	3.97E-04	0.00162	0.244	1
		Rhy - Ser	-1.23E-03	0.00162	-0.754	1
		Rhy - Sty	5.48E-03	0.00162	3.376	**0.0037**
		Sar - Ser	-1.62E-03	0.00162	-0.998	0.9542
		Sar - Sty	5.09E-03	0.00162	3.131	**0.0065**
		Ser - Sty	6.71E-03	0.00162	4.13	**0.0005**
	**N content of the host tissue (Figure 2A)**					
**GLM**			**Df**	**LR Chisq**	**Pr(>Chi)**	
		Depth	1	1.9564	0.1619	
		Species	5	16.0284	**0.0068**	
		Depth:Species	4	23.3805	**0.0001**	
**Emmeans**		**Pairwise comparison**	**estimate**	**SE**	**z.ratio**	**p.value**
	Gal	Shallow - Mesophotic	0.474	0.336	1.411	0.1584
	Rhy		-0.0177	0.336	-0.053	0.9580
	Sar		-0.3127	0.336	-0.931	0.3520
	Ser		1.4846	0.336	4.419	**<.0001**
	Sty		-0.5774	0.336	-1.718	0.0857
	Shallow	Gal - Lit	-0.5826	0.336	-1.734	0.1387
		Gal - Rhy	-0.4531	0.336	-1.348	0.2421
		Gal - Sar	0.129	0.336	0.384	0.8089
		Gal - Ser	0.7691	0.336	2.289	0.0828
		Gal - Sty	-0.5279	0.336	-1.571	0.1742
		Lit - Rhy	0.1296	0.336	0.386	0.8089
		Lit - Sar	0.7116	0.336	2.118	0.1025
		Lit - Ser	1.3517	0.336	4.023	**0.0008**
		Lit - Sty	0.0547	0.336	0.163	0.8706
		Rhy - Sar	0.582	0.336	1.732	0.1387
		Rhy - Ser	1.2221	0.336	3.637	**0.0014**
		Rhy - Sty	-0.0749	0.336	-0.223	0.8706
		Sar - Ser	0.6401	0.336	1.905	0.1216
		Sar - Sty	-0.6569	0.336	-1.955	0.1216
		Ser - Sty	-1.297	0.336	-3.86	**0.0008**
	Mesophotic	Gal - Rhy	0.0386	0.336	0.115	1.0000
		Gal - Sar	0.9156	0.336	2.725	**0.0452**
		Gal - Ser	-0.2416	0.336	-0.719	0.7868
		Gal - Sty	0.5234	0.336	1.558	0.3579
		Rhy - Sar	0.877	0.336	2.61	**0.0452**
		Rhy - Ser	-0.2802	0.336	-0.834	0.7581
		Rhy - Sty	0.4848	0.336	1.443	0.3726
		Sar - Ser	-1.1572	0.336	-3.444	**0.0086**
		Sar - Sty	-0.3922	0.336	-1.167	0.5208
		Ser - Sty	0.765	0.336	2.277	0.0855
	**N content of the Symbiodiniaceae fraction (Figure 2B)**					
**GLM**			**Df**	**LR Chisq**	**Pr(>Chi)**	
		Depth	1	5.57	**0.0183**	
		Species	5	398.3	**<.0001**	

		Depth:Species	4	7.6	0.1072	
**Emmeans**		**Pairwise comparison**	**estimate**	**SE**	**z.ratio**	**p.value**
	Gal	Shallow - Mesophotic	19.929	8.85	2.251	**0.0244**
	Rhy		-3.763	8.85	-0.425	0.6708
	Sar		-0.281	8.85	-0.032	0.9746
	Ser		23.956	8.85	2.706	**0.0068**
	Sty		6.885	8.85	0.778	0.4367
	Shallow	Gal - Lit	-4.75	8.85	-0.537	0.6337
		Gal - Rhy	50.78	8.85	5.736	**<.0001**
		Gal - Sar	23.55	8.85	2.66	**0.0090**
		Gal - Ser	-57.38	8.85	-6.482	**<.0001**
		Gal - Sty	-55.87	8.85	-6.311	**<.0001**
		Lit - Rhy	55.53	8.85	6.273	**<.0001**
		Lit - Sar	28.3	8.85	3.196	**0.0019**
		Lit - Ser	-52.63	8.85	-5.945	**<.0001**
		Lit - Sty	-51.12	8.85	-5.774	**<.0001**
		Rhy - Sar	-27.23	8.85	-3.076	**0.0026**
		Rhy - Ser	-108.16	8.85	-12.218	**<.0001**
		Rhy - Sty	-106.65	8.85	-12.047	**<.0001**
		Sar - Ser	-80.93	8.85	-9.142	**<.0001**
		Sar - Sty	-79.42	8.85	-8.971	**<.0001**
		Ser - Sty	1.51	8.85	0.171	0.8644
	Mesophotic	Gal - Rhy	27.09	8.85	3.06	**0.0047**
		Gal - Sar	3.34	8.85	0.377	1.0000
		Gal - Ser	-53.36	8.85	-6.027	**<.0001**
		Gal - Sty	-68.92	8.85	-7.785	**<.0001**
		Rhy - Sar	-23.75	8.85	-2.683	**0.0137**
		Rhy - Ser	-80.44	8.85	-9.087	**<.0001**
		Rhy - Sty	-96	8.85	-10.844	**<.0001**
		Sar - Ser	-56.69	8.85	-6.404	**<.0001**
		Sar - Sty	-72.25	8.85	-8.161	**<.0001**
		Ser - Sty	-15.56	8.85	-1.757	0.1314
	**C:N of the host tissue (Figure S2A)**					
**GLM**			**Df**	**LR Chisq**	**Pr(>Chi)**	
		Depth	1	1.45	0.2292	
		Species	5	798.95	**<.0001**	
		Depth:Species	4	5.9	0.2071	
**Emmeans**		**Pairwise comparison**	**estimate**	**SE**	**z.ratio**	**p.value**
	Gal	Shallow - Mesophotic	-0.0446	0.031	-1.435	0.1512
	Rhy		-0.0292	0.031	-0.94	0.3473
	Sar		0.0087	0.031	0.28	0.7793
	Ser		-0.0539	0.031	-1.736	0.0825
	Sty		0.0355	0.031	1.142	0.2533
	Shallow	Gal - Lit	0.12548	0.031	4.042	**0.0001**
		Gal - Rhy	-0.46568	0.031	-15	**<.0001**
		Gal - Sar	-0.09367	0.031	-3.017	**0.0038**

		Gal - Ser	-0.02532	0.031	-0.816	0.4148
		Gal - Sty	0.06681	0.031	2.152	**0.0362**
		Lit - Rhy	-0.59115	0.031	-19.041	**<.0001**
		Lit - Sar	-0.21914	0.031	-7.059	**<.0001**
		Lit - Ser	-0.15079	0.031	-4.857	**<.0001**
		Lit - Sty	-0.05867	0.031	-1.89	0.0630
		Rhy - Sar	0.37201	0.031	11.983	**<.0001**
		Rhy - Ser	0.44036	0.031	14.184	**<.0001**
		Rhy - Sty	0.53248	0.031	17.152	**<.0001**
		Sar - Ser	0.06835	0.031	2.202	**0.0346**
		Sar - Sty	0.16048	0.031	5.169	**<.0001**
		Ser - Sty	0.09213	0.031	2.967	**0.0041**
	Mesophotic	Gal - Rhy	-0.48106	0.031	-15.495	**<.0001**
		Gal - Sar	-0.14693	0.031	-4.733	**<.0001**
		Gal - Ser	-0.01598	0.031	-0.515	1.0000
		Gal - Sty	-0.01322	0.031	-0.426	1.0000
		Rhy - Sar	0.33413	0.031	10.762	**<.0001**
		Rhy - Ser	0.46508	0.031	14.98	**<.0001**
		Rhy - Sty	0.46784	0.031	15.069	**<.0001**
		Sar - Ser	0.13095	0.031	4.218	**0.0001**
		Sar - Sty	0.13371	0.031	4.307	**<.0001**
		Ser - Sty	0.00276	0.031	0.089	1.0000
	**C:N of the Symbiodiniaceae fraction (Figure S2B)**					
**GLM**			**Df**	**LR Chisq**	**Pr(>Chi)**	
		Depth	1	0.794	0.3729	
		Species	5	255.324	**<.0001**	
		Depth:Species	4	5.000	0.2873	
**Emmeans**		**Pairwise comparison**	**estimate**	**SE**	**z.ratio**	**p.value**
	Gal	Shallow - Mesophotic	-0.01024	0.00813	-1.26	0.2078
	Rhy		0.01213	0.00813	1.492	0.1356
	Sar		-0.00624	0.00813	-0.767	0.4429
	Ser		-0.00922	0.00813	-1.134	0.2566
	Sty		-0.00263	0.00813	-0.323	0.7463
	Shallow	Gal - Lit	0.07239	0.00813	8.905	**<.0001**
		Gal - Rhy	-0.03914	0.00813	-4.815	**<.0001**
		Gal - Sar	0.03111	0.00813	3.828	**0.0002**
		Gal - Ser	0.01796	0.00813	2.209	**0.0339**
		Gal - Sty	0.02229	0.00813	2.742	**0.0083**
		Lit - Rhy	-0.11152	0.00813	-13.721	**<.0001**
		Lit - Sar	-0.04127	0.00813	-5.077	**<.0001**
		Lit - Ser	-0.05443	0.00813	-6.696	**<.0001**
		Lit - Sty	-0.0501	0.00813	-6.164	**<.0001**
		Rhy - Sar	0.07025	0.00813	8.643	**<.0001**
		Rhy - Ser	0.0571	0.00813	7.024	**<.0001**
		Rhy - Sty	0.06142	0.00813	7.557	**<.0001**
		Sar - Ser	-0.01316	0.00813	-1.619	0.1217

		Sar - Sty	-0.00883	0.00813	-1.086	0.2972
		Ser - Sty	0.00433	0.00813	0.532	0.5944
	Mesophotic	Gal - Rhy	-0.01677	0.00813	-2.063	0.0838
		Gal - Sar	0.03512	0.00813	4.32	**0.0001**
		Gal - Ser	0.01898	0.00813	2.334	**0.0489**
		Gal - Sty	0.02989	0.00813	3.678	**0.0007**
		Rhy - Sar	0.05189	0.00813	6.384	**<.0001**
		Rhy - Ser	0.03575	0.00813	4.398	**0.0001**
		Rhy - Sty	0.04666	0.00813	5.741	**<.0001**
		Sar - Ser	-0.01614	0.00813	-1.986	0.0882
		Sar - Sty	-0.00522	0.00813	-0.642	0.7808
		Ser - Sty	0.01092	0.00813	1.343	0.2986
	**C content of the host tissue (Figure S2C)**					
**GLM**			**Df**	**LR Chisq**	**Pr(>Chi)**	
		Depth	1	0.26	0.6107	
		Species	5	474.95	**<.0001**	
		Depth:Species	4	18.44	**0.0010**	
**Emmeans**		**Pairwise comparison**	**estimate**	**SE**	**z.ratio**	**p.value**
	Gal	Shallow - Mesophotic	47	43.5	1.081	0.2795
	Rhy		-69.6	43.5	-1.601	0.1095
	Sar		-36.2	43.5	-0.833	0.4050
	Ser		156.9	43.5	3.609	**0.0003**
	Sty		-48.6	43.5	-1.119	0.2633
	Shallow	Gal - Lit	-20.799	43.5	-0.478	0.7727
		Gal - Rhy	-575.204	43.5	-13.227	**<.0001**
		Gal - Sar	-25.964	43.5	-0.597	0.7506
		Gal - Ser	64.276	43.5	1.478	0.2323
		Gal - Sty	-39.35	43.5	-0.905	0.5483
		Lit - Rhy	-554.405	43.5	-12.749	**<.0001**
		Lit - Sar	-5.165	43.5	-0.119	0.9055
		Lit - Ser	85.076	43.5	1.956	0.0946
		Lit - Sty	-18.551	43.5	-0.427	0.7727
		Rhy - Sar	549.24	43.5	12.63	**<.0001**
		Rhy - Ser	639.481	43.5	14.705	**<.0001**
		Rhy - Sty	535.854	43.5	12.322	**<.0001**
		Sar - Ser	90.241	43.5	2.075	0.0814
		Sar - Sty	-13.386	43.5	-0.308	0.8124
		Ser - Sty	-103.627	43.5	-2.383	**0.0429**
	Mesophotic	Gal - Rhy	-458.567	43.5	-10.545	**<.0001**
		Gal - Sar	57.277	43.5	1.317	0.3661
		Gal - Ser	-45.629	43.5	-1.049	0.4901
		Gal - Sty	56.322	43.5	1.295	0.3661
		Rhy - Sar	515.844	43.5	11.862	**<.0001**
		Rhy - Ser	412.938	43.5	9.496	**<.0001**
		Rhy - Sty	514.889	43.5	11.84	**<.0001**
		Sar - Ser	-102.906	43.5	-2.366	**0.0476**

		Sar - Sty	-0.955	43.5	-0.022	1.0000
		Ser - Sty	101.951	43.5	2.344	**0.0476**
	**C content of the Symbiodiniaceae fraction (Figure S2D)**					
**GLM**			**Df**	**LR Chisq**	**Pr(>Chi)**	
		Depth	1	8.54	**0.0035**	
		Species	5	705.97	**<.0001**	
		Depth:Species	4	7.58	0.1081	
**Emmeans**		**Pairwise comparison**	**estimate**	**SE**	**z.ratio**	**p.value**
	Gal	Shallow - Mesophotic	3.216	1.2	2.674	**0.0075**
	Rhy		2.315	1.2	1.925	0.0543
	Sar		-0.863	1.2	-0.718	0.4730
	Ser		2.541	1.2	2.113	**0.0346**
	Sty		0.652	1.2	0.542	0.5876
	Shallow	Gal - Lit	8.357	1.2	6.948	**<.0001**
		Gal - Rhy	6.076	1.2	5.051	**<.0001**
		Gal - Sar	10.648	1.2	8.853	**<.0001**
		Gal - Ser	-7.625	1.2	-6.339	**<.0001**
		Gal - Sty	-6.531	1.2	-5.43	**<.0001**
		Lit - Rhy	-2.281	1.2	-1.897	0.0620
		Lit - Sar	2.291	1.2	1.905	0.0620
		Lit - Ser	-15.982	1.2	-13.287	**<.0001**
		Lit - Sty	-14.888	1.2	-12.377	**<.0001**
		Rhy - Sar	4.572	1.2	3.802	**0.0002**
		Rhy - Ser	-13.7	1.2	-11.391	**<.0001**
		Rhy - Sty	-12.606	1.2	-10.481	**<.0001**
		Sar - Ser	-18.273	1.2	-15.192	**<.0001**
		Sar - Sty	-17.179	1.2	-14.282	**<.0001**
		Ser - Sty	1.094	1.2	0.91	0.3630
	Mesophotic	Gal - Rhy	5.175	1.2	4.302	**<.0001**
		Gal - Sar	6.569	1.2	5.462	**<.0001**
		Gal - Ser	-8.299	1.2	-6.9	**<.0001**
		Gal - Sty	-9.094	1.2	-7.561	**<.0001**
		Rhy - Sar	1.395	1.2	1.159	0.4105
		Rhy - Ser	-13.474	1.2	-11.202	**<.0001**
		Rhy - Sty	-14.269	1.2	-11.863	**<.0001**
		Sar - Ser	-14.868	1.2	-12.362	**<.0001**
		Sar - Sty	-15.663	1.2	-13.022	**<.0001**
		Ser - Sty	-0.795	1.2	-0.661	0.7630

Table S3: Statistical analysis for the assimilation rates. *Galaxea fascicularis* (Gal), *Seriatopora hystrix* (Ser), *Stylophora pistillata* (Sty) are hard corals and *Litophyton arboreum* (Lit), *Rhytisma fulvum fulvum* (Rhy), *Sarcophyton* sp. (Sar) are soft corals. Significant differences are displayed in bold. DFAA = dissolved free amino acids.

	Assimilation of DN in the host tissue (Figure 3)						
**GLM**				**Df**	**LR Chisq**	**Pr(>Chi)**	
			Depth	1	42.54	**<.0001**	
			Species	5	3133.55	**<.0001**	
			Source	2	287.1	**<.0001**	
			Species:Depth	4	17.91	**0.0013**	
			Species:Source	10	209.46	**<.0001**	
			Depth:Source	2	23.08	**<.0001**	
			Species:Depth:Source	8	22.21	**0.0045**	
**Emmeans**			**Pairwise comparison**	**estimate**	**SE**	**z.ratio**	**p.value**
	Ammonium	Gal	Shallow - Mesophotic	-0.054	0.236	-0.230	0.8180
		Rhy		0.328	0.250	1.310	0.1902
		Sar		-0.135	0.250	-0.538	0.5902
		Ser		-0.009	0.236	-0.036	0.9710
		Sty		-0.055	0.236	-0.235	0.8145
	Nitrate	Gal		-0.229	0.236	-0.970	0.3322
		Rhy		0.714	0.236	3.025	**0.0025**
		Sar		0.244	0.236	1.035	0.3008
		Ser		0.720	0.236	3.051	**0.0023**
		Sty		0.796	0.236	3.373	**0.0007**
	DFAA	Gal		0.902	0.236	3.824	**0.0001**
		Rhy		0.413	0.236	1.749	0.0803
		Sar		-0.126	0.236	-0.534	0.5930
		Ser		1.313	0.236	5.563	**<.0001**
		Sty		1.116	0.236	4.730	**<.0001**
	Ammonium	Shallow	Gal - Lit	2.026	0.250	8.093	**<.0001**
			Gal - Rhy	1.817	0.250	7.258	**<.0001**
			Gal - Sar	3.264	0.250	13.039	**<.0001**
			Gal - Ser	-0.245	0.236	-1.037	0.3747
			Gal - Sty	-0.076	0.236	-0.323	0.7467
			Lit - Rhy	-0.209	0.264	-0.792	0.4944
			Lit - Sar	1.238	0.264	4.692	**<.0001**
			Lit - Ser	-2.271	0.250	-9.071	**<.0001**
			Lit - Sty	-2.102	0.250	-8.397	**<.0001**
			Rhy - Sar	1.447	0.264	5.484	**<.0001**
			Rhy - Ser	-2.062	0.250	-8.236	**<.0001**
			Rhy - Sty	-1.893	0.250	-7.563	**<.0001**
			Sar - Ser	-3.509	0.250	-14.017	**<.0001**
			Sar - Sty	-3.340	0.250	-13.344	**<.0001**
			Ser - Sty	0.169	0.236	0.714	0.5092
		Mesophotic	Gal - Rhy	1.435	0.236	6.079	**<.0001**

		Gal - Sar	3.345	0.236	14.171	**<.0001**
		Gal - Ser	-0.290	0.236	-1.231	0.4095
		Gal - Sty	-0.075	0.236	-0.318	1.0000
		Rhy - Sar	1.910	0.236	8.092	**<.0001**
		Rhy - Ser	-1.725	0.236	-7.310	**<.0001**
		Rhy - Sty	-1.510	0.236	-6.397	**<.0001**
		Sar - Ser	-3.635	0.236	-15.402	**<.0001**
		Sar - Sty	-3.420	0.236	-14.490	**<.0001**
		Ser - Sty	0.215	0.236	0.912	0.6026
Nitrate	Shallow	Gal - Lit	4.496	0.236	19.049	**<.0001**
		Gal - Rhy	2.788	0.236	11.813	**<.0001**
		Gal - Sar	4.194	0.236	17.770	**<.0001**
		Gal - Ser	0.895	0.236	3.794	**0.0002**
		Gal - Sty	1.289	0.236	5.461	**<.0001**
		Lit - Rhy	-1.708	0.236	-7.236	**<.0001**
		Lit - Sar	-0.302	0.236	-1.279	0.2010
		Lit - Ser	-3.600	0.236	-15.255	**<.0001**
		Lit - Sty	-3.207	0.236	-13.588	**<.0001**
		Rhy - Sar	1.406	0.236	5.957	**<.0001**
		Rhy - Ser	-1.893	0.236	-8.019	**<.0001**
		Rhy - Sty	-1.499	0.236	-6.353	**<.0001**
		Sar - Ser	-3.299	0.236	-13.976	**<.0001**
		Sar - Sty	-2.905	0.236	-12.310	**<.0001**
		Ser - Sty	0.393	0.236	1.667	0.1024
	Mesophotic	Gal - Rhy	1.845	0.236	7.818	**<.0001**
		Gal - Sar	3.721	0.236	15.766	**<.0001**
		Gal - Ser	-0.054	0.236	-0.227	1.0000
		Gal - Sty	0.264	0.236	1.117	0.4398
		Rhy - Sar	1.876	0.236	7.948	**<.0001**
		Rhy - Ser	-1.899	0.236	-8.045	**<.0001**
		Rhy - Sty	-1.581	0.236	-6.701	**<.0001**
		Sar - Ser	-3.774	0.236	-15.992	**<.0001**
		Sar - Sty	-3.457	0.236	-14.649	**<.0001**
		Ser - Sty	0.317	0.236	1.344	0.3356
DFAA	Shallow	Gal - Lit	2.684	0.236	11.371	**<.0001**
		Gal - Rhy	3.000	0.236	12.712	**<.0001**
		Gal - Sar	4.856	0.236	20.576	**<.0001**
		Gal - Ser	0.441	0.236	1.868	0.0771
		Gal - Sty	0.251	0.236	1.065	0.3075
		Lit - Rhy	0.317	0.236	1.342	0.2074
		Lit - Sar	2.173	0.236	9.206	**<.0001**
		Lit - Ser	-2.243	0.236	-9.502	**<.0001**
		Lit - Sty	-2.432	0.236	-10.306	**<.0001**
		Rhy - Sar	1.856	0.236	7.864	**<.0001**
		Rhy - Ser	-2.559	0.236	-10.844	**<.0001**
		Rhy - Sty	-2.749	0.236	-11.647	**<.0001**

			Sar - Ser	-4.415	0.236	-18.708	**<.0001**
			Sar - Sty	-4.605	0.236	-19.512	**<.0001**
			Ser - Sty	-0.190	0.236	-0.804	0.4216
		Mesophotic	Gal - Rhy	3.490	0.236	14.787	**<.0001**
			Gal - Sar	5.885	0.236	24.934	**<.0001**
			Gal - Ser	0.030	0.236	0.129	1.0000
			Gal - Sty	0.037	0.236	0.159	1.0000
			Rhy - Sar	2.395	0.236	10.148	**<.0001**
			Rhy - Ser	-3.459	0.236	-14.658	**<.0001**
			Rhy - Sty	-3.452	0.236	-14.628	**<.0001**
			Sar - Ser	-5.854	0.236	-24.805	**<.0001**
			Sar - Sty	-5.847	0.236	-24.776	**<.0001**
			Ser - Sty	0.007	0.236	0.030	1.0000
	**Assimilation of DN in the Symbiodiniaceae fraction (Figure 4)**						
**GLM**				**Df**	**LR Chisq**	**Pr(>Chi)**	
			Depth	1	0.65	0.4213	
			Species	5	1377.93	**<.0001**	
			Source	2	122.91	**<.0001**	
			Species:Depth	4	9.57	**0.0484**	
			Species:Source	10	131.63	**<.0001**	
			Depth:Source	2	20.48	**<.0001**	
			Species:Depth:Source	8	16.92	**0.0310**	
**Emmeans**			**Pairwise comparison**	**estimate**	**SE**	**z.ratio**	**p.value**
	Ammonium	Gal	Shallow - Mesophotic	-0.455	0.281	-1.619	0.1055
		Rhy		0.446	0.298	1.495	0.1350
		Sar		-0.756	0.281	-2.687	**0.0072**
		Ser		-0.085	0.281	-0.302	0.7630
		Sty		-0.371	0.281	-1.320	0.1867
	Nitrate	Gal		-0.671	0.281	-2.388	**0.0170**
		Rhy		-0.699	0.298	-2.345	**0.0190**
		Sar		-0.692	0.281	-2.460	**0.0139**
		Ser		0.180	0.298	0.602	0.5472
		Sty		-0.243	0.314	-0.772	0.4398
	DFAA	Gal		0.465	0.298	1.560	0.1188
		Rhy		0.886	0.281	3.149	**0.0016**
		Sar		0.202	0.281	0.719	0.4719
		Ser		-0.248	0.314	-0.789	0.4299
		Sty		0.792	0.281	2.817	**0.0049**
	Ammonium	Shallow	Gal - Lit	0.588	0.281	2.089	**0.0423**
			Gal - Rhy	1.483	0.298	4.973	**<.0001**
			Gal - Sar	2.347	0.281	8.346	**<.0001**
			Gal - Ser	-0.563	0.281	-2.004	**0.0483**
			Gal - Sty	-0.795	0.281	-2.826	**0.0059**
			Lit - Rhy	0.896	0.298	3.004	**0.0040**
			Lit - Sar	1.759	0.281	6.257	**<.0001**

		Lit - Ser	-1.151	0.281	-4.093	**0.0001**
		Lit - Sty	-1.382	0.281	-4.915	**<.0001**
		Rhy - Sar	0.864	0.298	2.895	**0.0052**
		Rhy - Ser	-2.047	0.298	-6.862	**<.0001**
		Rhy - Sty	-2.278	0.298	-7.638	**<.0001**
		Sar - Ser	-2.910	0.281	-10.350	**<.0001**
		Sar - Sty	-3.142	0.281	-11.172	**<.0001**
		Ser - Sty	-0.231	0.281	-0.822	0.4110
	Mesophotic	Gal - Rhy	0.582	0.281	2.071	0.0639
		Gal - Sar	2.647	0.281	9.414	**<.0001**
		Gal - Ser	-0.934	0.281	-3.321	**0.0019**
		Gal - Sty	-0.879	0.281	-3.124	**0.0033**
		Rhy - Sar	2.065	0.281	7.343	**<.0001**
		Rhy - Ser	-1.516	0.281	-5.392	**<.0001**
		Rhy - Sty	-1.461	0.281	-5.195	**<.0001**
		Sar - Ser	-3.581	0.281	-12.735	**<.0001**
		Sar - Sty	-3.526	0.281	-12.539	**<.0001**
		Ser - Sty	0.055	0.281	0.197	1.0000
Nitrate	Shallow	Gal - Lit	3.639	0.281	12.942	**<.0001**
		Gal - Rhy	1.777	0.298	5.957	**<.0001**
		Gal - Sar	3.533	0.281	12.563	**<.0001**
		Gal - Ser	-0.173	0.298	-0.581	0.6477
		Gal - Sty	-0.232	0.298	-0.779	0.5452
		Lit - Rhy	-1.863	0.298	-6.245	**<.0001**
		Lit - Sar	-0.107	0.281	-0.380	0.7545
		Lit - Ser	-3.813	0.298	-12.783	**<.0001**
		Lit - Sty	-3.872	0.298	-12.981	**<.0001**
		Rhy - Sar	1.756	0.298	5.887	**<.0001**
		Rhy - Ser	-1.950	0.314	-6.203	**<.0001**
		Rhy - Sty	-2.009	0.314	-6.390	**<.0001**
		Sar - Ser	-3.706	0.298	-12.425	**<.0001**
		Sar - Sty	-3.765	0.298	-12.623	**<.0001**
		Ser - Sty	-0.059	0.314	-0.188	0.8511
	Mesophotic	Gal - Rhy	1.805	0.281	6.418	**<.0001**
		Gal - Sar	3.553	0.281	12.634	**<.0001**
		Gal - Ser	-1.024	0.281	-3.642	**0.0005**
		Gal - Sty	-0.661	0.298	-2.216	**0.0445**
		Rhy - Sar	1.748	0.281	6.216	**<.0001**
		Rhy - Ser	-2.829	0.281	-10.061	**<.0001**
		Rhy - Sty	-2.466	0.298	-8.267	**<.0001**
		Sar - Ser	-4.577	0.281	-16.277	**<.0001**
		Sar - Sty	-4.214	0.298	-14.128	**<.0001**
		Ser - Sty	0.363	0.298	1.218	0.3346
DFAA	Shallow	Gal - Lit	0.322	0.281	1.146	0.2519
		Gal - Rhy	2.349	0.281	8.355	**<.0001**
		Gal - Sar	3.013	0.281	10.716	**<.0001**

			Gal - Ser	-0.686	0.298	-2.300	**0.0268**
			Gal - Sty	-0.325	0.281	-1.157	0.2519
			Lit - Rhy	2.027	0.281	7.209	**<.0001**
			Lit - Sar	2.691	0.281	9.570	**<.0001**
			Lit - Ser	-1.008	0.298	-3.381	**0.0012**
			Lit - Sty	-0.647	0.281	-2.302	**0.0268**
			Rhy - Sar	0.664	0.281	2.361	**0.0268**
			Rhy - Ser	-3.035	0.298	-10.177	**<.0001**
			Rhy - Sty	-2.675	0.281	-9.511	**<.0001**
			Sar - Ser	-3.699	0.298	-12.403	**<.0001**
			Sar - Sty	-3.339	0.281	-11.873	**<.0001**
			Ser - Sty	0.361	0.298	1.210	0.2519
		Mesophotic	Gal - Rhy	1.929	0.298	6.468	**<.0001**
			Gal - Sar	3.276	0.298	10.985	**<.0001**
			Gal - Ser	0.027	0.314	0.087	1.0000
			Gal - Sty	-0.652	0.298	-2.186	**0.0480**
			Rhy - Sar	1.347	0.281	4.791	**<.0001**
			Rhy - Ser	-1.902	0.298	-6.376	**<.0001**
			Rhy - Sty	-2.581	0.281	-9.179	**<.0001**
			Sar - Ser	-3.249	0.298	-10.893	**<.0001**
			Sar - Sty	-3.928	0.281	-13.970	**<.0001**
			Ser - Sty	-0.679	0.298	-2.278	**0.0426**
	**Assimilation of DN in the host tissue of soft corals exposed at two temperatures (Figure 5A)**						
**GLM**				**Df**	**LR Chisq**	**Pr(>Chi)**	
			Temperature	1	18.72	**<.0001**	
			Species	2	298.628	**<.0001**	
			Source	2	290.587	**<.0001**	
			Species:Temperature	2	11.24	**0.0036**	
			Species:Source	4	76.78	**<.0001**	
			Temperature:Source	2	3.785	0.1507	
			Species:Temperature:Source	4	11.095	**0.0255**	
**Emmeans**			**Pairwise comparison**	**estimate**	**SE**	**z.ratio**	**p.value**
	Ammonium	Lit	25°C - 30°C	-0.885	0.262	-3.381	**0.0007**
		Rhy		-0.639	0.278	-2.304	**0.0212**
		Sar		0.499	0.293	1.705	**0.0881**
	Nitrate	Lit		-0.223	0.262	-0.851	0.3948
		Rhy		-0.558	0.278	-2.012	**0.0442**
		Sar		0.246	0.262	0.940	0.3472
	DFAA	Lit		-0.211	0.262	-0.805	0.4207
		Rhy		-0.968	0.262	-3.701	**0.0002**
		Sar		-0.610	0.262	-2.330	**0.0198**
	Ammonium	25°C	Lit - Rhy	-0.537	0.278	-1.935	**0.0529**
			Lit - Sar	0.955	0.278	3.440	**0.0009**

			Rhy - Sar	1.492	0.293	5.099	**<.0001**
		30°C	Lit - Rhy	-0.292	0.262	-1.115	0.2649
			Lit - Sar	2.338	0.278	8.425	**<.0001**
			Rhy - Sar	2.630	0.278	9.476	**<.0001**
	Nitrate	25°C	Lit - Rhy	-1.673	0.262	-6.393	**<.0001**
			Lit - Sar	-0.288	0.262	-1.100	0.2715
			Rhy - Sar	1.385	0.262	5.293	**<.0001**
		30°C	Lit - Rhy	-2.008	0.278	-7.237	**<.0001**
			Lit - Sar	0.181	0.262	0.691	0.4893
			Rhy - Sar	2.189	0.278	7.889	**<.0001**
	DFAA	25°C	Lit - Rhy	0.310	0.262	1.183	0.2366
			Lit - Sar	2.066	0.262	7.897	**<.0001**
			Rhy - Sar	1.757	0.262	6.714	**<.0001**
		30°C	Lit - Rhy	-0.448	0.262	-1.712	0.0868
			Lit - Sar	1.667	0.262	6.373	**<.0001**
			Rhy - Sar	2.115	0.262	8.085	**<.0001**
	**Assimilation of DN in the Symbiodiniaceae fraction of soft corals exposed at two temperatures (Figure 5B)**						
**GLM**				**Df**	**LR Chisq**	**Pr(>Chi)**	
			Temperature	1	8.127	**0.0044**	
			Species	2	145.908	**<.0001**	
			Source	2	144.296	**<.0001**	
			Species:Temperature	2	11.792	**0.0028**	
			Species:Source	4	169.734	**<.0001**	
			Temperature:Source	2	1.524	0.4667	
			Species:Temperature:Source	4	3.153	0.5326	
**Emmeans**			**Pairwise comparison**	**estimate**	**SE**	**z.ratio**	**p.value**
	Ammonium	Lit	25°C - 30°C	0.153	0.338	0.453	0.6509
		Rhy		-0.742	0.310	-2.392	**0.0168**
		Sar		-0.030	0.292	-0.103	0.9183
	Nitrate	Lit		0.071	0.310	0.229	0.8186
		Rhy		-0.727	0.310	-2.342	**0.0192**
		Sar		0.036	0.292	0.124	0.9014
	DFAA	Lit		0.144	0.292	0.492	0.6228
		Rhy		-0.742	0.310	-2.391	**0.0168**
		Sar		-0.766	0.292	-2.619	**0.0088**
	Ammonium	25°C	Lit - Rhy	0.896	0.310	2.888	**0.0054**
			Lit - Sar	1.759	0.292	6.015	**<.0001**
			Rhy - Sar	0.864	0.310	2.783	**0.0054**
		30°C	Lit - Rhy	0.001	0.338	0.003	0.9979
			Lit - Sar	1.577	0.338	4.668	**<.0001**
			Rhy - Sar	1.576	0.292	5.387	**<.0001**
	Nitrate	25°C	Lit - Rhy	-1.845	0.327	-5.641	**<.0001**
			Lit - Sar	-0.241	0.310	-0.778	0.4367

		Rhy - Sar	1.603	0.310	5.168	**<.0001**
	30°C	Lit - Rhy	-2.643	0.292	-9.035	**<.0001**
		Lit - Sar	-0.276	0.292	-0.944	0.3450
		Rhy - Sar	2.366	0.292	8.090	**<.0001**
DFAA	25°C	Lit - Rhy	2.027	0.292	6.930	**<.0001**
		Lit - Sar	2.691	0.292	9.200	**<.0001**
		Rhy - Sar	0.664	0.292	2.270	**0.0232**
	30°C	Lit - Rhy	1.141	0.310	3.679	**0.0004**
		Lit - Sar	1.781	0.292	6.090	**<.0001**
		Rhy - Sar	0.640	0.310	2.063	**0.0391**
**Assimilation of DN in the host tissue of hard corals, normalized to surface area of the skeleton (Figure S3A)**						
**GLM**			**Df**	**LR Chisq**	**Pr(>Chi)**	
		Depth	1	0.146	0.7024	
		Species	2	123.448	**<.0001**	
		Source	2	218.803	**<.0001**	
		Species:Depth	2	35.297	**<.0001**	
		Species:Source	4	63.225	**<.0001**	
		Depth:Source	2	18.866	**<.0001**	
		Species:Depth:Source	4	10.643	**0.0309**	
**Emmeans**		**Pairwise comparison**	**estimate**	**SE**	**z.ratio**	**p.value**
Ammonium	Gal	Shallow - Mesophotic	1.211	0.635	1.907	0.0566
	Ser		1.703	0.635	2.681	**0.0073**
	Sty		-0.802	0.635	-1.263	0.2066
Nitrate	Gal		2.826	0.599	4.718	**<.0001**
	Ser		0.255	0.599	0.426	0.6699
	Sty		-1.025	0.692	-1.483	0.1382
DFAA	Gal		0.949	0.635	1.494	0.1352
	Ser		-2.298	0.635	-3.618	**0.0003**
	Sty		-2.335	0.599	-3.899	**0.0001**
Ammonium	Shallow	Gal - Ser	-2.332	0.635	-3.671	**0.0004**
		Galax - Sty	2.076	0.635	3.268	**0.0011**
		Ser - Sty	4.408	0.599	7.360	**<.0001**
	Mesophotic	Gal - Ser	-1.840	0.635	-2.896	**0.0067**
		Galax - Sty	0.063	0.635	0.098	0.9217
		Ser - Sty	1.902	0.670	2.841	**0.0067**
Nitrate	Shallow	Gal - Ser	3.799	0.599	6.343	**<.0001**
		Galax - Sty	6.245	0.599	10.427	**<.0001**
		Ser - Sty	2.446	0.599	4.084	**<.0001**
	Mesophotic	Gal - Ser	1.229	0.599	2.051	0.0604
		Galax - Sty	2.394	0.692	3.462	**0.0016**
		Ser - Sty	1.166	0.692	1.685	0.0919
DFAA	Shallow	Gal - Ser	1.060	0.670	1.582	0.1135
		Galax - Sty	3.392	0.635	5.339	**<.0001**
		Ser - Sty	2.332	0.635	3.671	**0.0004**

		Mesophotic	Gal - Ser	-2.188	0.599	-3.653	**0.0004**
			Galax - Sty	0.108	0.599	0.180	0.8572
			Ser - Sty	2.296	0.599	3.833	**0.0004**
	**Assimilation of DN in the Symbiodiniaceae fraction of hard corals, normalized to surface area of the skeleton (Figure S3B)**						
**GLM**				**Df**	**LR Chisq**	**Pr(>Chi)**	
			Depth	1	32.315	**<.0001**	
			Species	2	40.566	**<.0001**	
			Source	2	37.361	**<.0001**	
			Species:Depth	2	7.896	**0.0193**	
			Species:Source	4	10.312	**0.0356**	
			Depth:Source	2	0.937	0.6258	
			Species:Depth:Source	4	10.223	**0.0368**	
**Emmeans**			**Pairwise comparison**	**estimate**	**SE**	**z.ratio**	**p.value**
	Ammonium	Gal	Shallow - Mesophotic	0.751	0.412	1.822	0.0684
		Ser		0.368	0.412	0.893	0.3716
		Sty		0.899	0.412	2.181	**0.0292**
	Nitrate	Gal		1.650	0.412	4.002	**0.0001**
		Ser		1.008	0.437	2.305	**0.0212**
		Sty		0.293	0.437	0.669	0.5035
	DFAA	Gal		1.209	0.437	2.764	**0.0057**
		Ser		1.532	0.437	3.502	**0.0005**
		Sty		-0.405	0.412	-0.982	0.3259
	Ammonium	Shallow	Gal - Ser	-1.594	0.412	-3.866	**0.0003**
			Galax - Sty	-0.894	0.412	-2.168	**0.0453**
			Ser - Sty	0.700	0.412	1.699	0.0893
		Mesophotic	Gal - Ser	-1.977	0.412	-4.795	**<.0001**
			Galax - Sty	-0.746	0.412	-1.809	0.0705
			Ser - Sty	1.231	0.412	2.986	**0.0042**
	Nitrate	Shallow	Gal - Ser	-0.407	0.437	-0.929	0.3526
			Galax - Sty	0.734	0.412	1.780	0.1126
			Ser - Sty	1.141	0.437	2.608	**0.0273**
		Mesophotic	Gal - Ser	-1.049	0.412	-2.543	**0.0330**
			Galax - Sty	-0.623	0.437	-1.425	0.2310
			Ser - Sty	0.425	0.437	0.972	0.3311
	DFAA	Shallow	Gal - Ser	-0.762	0.412	-1.848	0.0799
			Galax - Sty	0.722	0.412	1.751	0.0799
			Ser - Sty	1.484	0.412	3.599	**0.0010**
		Mesophotic	Gal - Ser	-0.440	0.461	-0.954	0.3403
			Galax - Sty	-0.892	0.437	-2.040	0.1242
			Ser - Sty	-0.452	0.437	-1.034	0.3403

Translocation percentages from the Symbiodiniaceae to the host fraction (Table 1)							
**GLM**				**Df**	**LR Chisq**	**Pr(>Chi)**	
	25°C	N-NH_4^+	Species	5	54.699	**<.0001**	
			Depth	1	4.867	**0.0274**	
			Species:Depth	4	3.666	0.4530	
		N-NO_3^-	Species	5	31.3372	**<.0001**	
			Depth	1	24.7757	**<.0001**	
			Species:Depth	4	3.0132	0.5556	
	30°C	N-NH_4^+	Species	2	11.9962	**0.0025**	
			Temperature	1	0.0298	0.8631	
			Species: Temperature	2	4.4335	0.1090	
		N-NO_3^-	Species	2	0.7411	0.6903	
			Temperature	1	0.8793	0.3484	
			Species: Temperature	2	3.4749	0.1760	
**Emmeans**			**Pairwise comparison**	**estimate**	**SE**	**z.ratio**	**p.value**
	N-NH_4^+	Gal	Shallow - Mesophotic	307.6	180	1.710	0.0873
		Rhy		-77.6	191	-0.407	0.6840
		Sar		347.9	191	1.824	0.0682
		Ser		66.3	180	0.369	0.7123
		Sty		253.2	180	1.408	0.1591
	N-NO_3^-	Gal		623	430	1.449	0.1472
		Rhy		1503	430	3.494	**0.0005**
		Sar		1226	430	2.850	**0.0044**
		Ser		750	456	1.643	0.1003
		Sty		744	456	1.631	0.1028
	N-NH_4^+	Shallow	Gal - Lit	1011.5	180	5.624	**<.0001**
			Gal - Rhy	299.6	191	1.57	0.1586
			Gal - Sar	664.3	191	3.482	**0.0019**
			Gal - Ser	220.7	180	1.227	0.2536
			Gal - Sty	550.7	180	3.062	**0.0066**
			Lit - Rhy	-711.9	191	-3.732	**0.0001**
			Lit - Sar	-347.2	191	-1.82	0.1046
			Lit - Ser	-790.8	180	-4.397	**0.0001**
			Lit - Sty	-460.8	180	-2.562	**0.0260**
			Rhy - Sar	364.7	201	1.814	0.1046
			Rhy - Ser	-78.9	191	-0.414	0.6792
			Rhy - Sty	251.1	191	1.316	0.2351
			Sar - Ser	-443.6	191	-2.325	**0.0430**
			Sar - Sty	-113.6	191	-0.596	0.5909
			Ser - Sty	330	180	1.835	0.1046
		Mesophotic	Gal - Rhy	684.8	180	3.807	**0.0021**
			Gal - Sar	624	180	3.469	**0.0038**
			Gal - Ser	461.9	180	2.568	**0.0383**
			Gal - Sty	605	180	3.364	**0.0038**

			Rhy - Sar	-60.8	180	-0.338	1.0000
			Rhy - Ser	-222.9	180	-1.239	0.6458
			Rhy - Sty	-79.8	180	-0.444	1.0000
			Sar - Ser	-162	180	-0.901	0.9135
			Sar - Sty	-19	180	-0.105	1.0000
			Ser - Sty	143.1	180	0.796	0.9135
			Gal - Lit	1402.2	430	3.26	**0.0056**
			Gal - Rhy	1135.6	430	2.64	**0.0248**
			Gal - Sar	1180.9	430	2.746	**0.0226**
			Gal - Ser	1554.6	456	3.408	**0.0049**
			Gal - Sty	1468.3	430	3.414	**0.0049**
			Lit - Rhy	-266.6	430	-0.62	0.8031
			Lit - Sar	-221.3	430	-0.515	0.8276
		Shallow	Lit - Ser	152.4	456	0.334	0.9161
			Lit - Sty	66.1	430	0.154	0.9161
			Rhy - Sar	45.3	430	0.105	0.9161
			Rhy - Ser	419	456	0.918	0.8031
			Rhy - Sty	332.7	430	0.774	0.8031
	$N\text{-}NO_3^-$		Sar - Ser	373.7	456	0.819	0.8031
			Sar - Sty	287.4	430	0.668	0.8031
			Ser - Sty	-86.3	456	-0.189	0.9161
			Gal - Rhy	256.3	430	0.596	0.9187
			Gal - Sar	578.4	430	1.345	0.3828
			Gal - Ser	1428.3	430	3.321	**0.0135**
			Gal - Sty	1347.4	456	2.954	**0.0236**
		Mesophotic	Rhy - Sar	322.1	430	0.749	0.8510
			Rhy - Ser	1172	430	2.725	**0.0322**
			Rhy - Sty	1091.1	456	2.392	0.0629
			Sar - Ser	849.8	430	1.976	0.1445
			Sar - Sty	768.9	456	1.686	0.2297
			Ser - Sty	-80.9	456	-0.177	1.0000
		Lit		-43.5	23.7	-1.835	0.0665
	$N\text{-}NH_4^+$	Rhy		9.8	21.8	0.450	0.6529
		Sar	25°C – 30°C	20.6	21.8	0.945	0.3446
		Lit		-22.7	23.2	-0.980	0.3271
	$N\text{-}NO_3^-$	Rhy		31.0	23.2	1.339	0.1896
		Sar		29.3	23.2	1.265	0.2057
			Lit - Rhy	-21.8	23.7	-0.917	0.3798
		$N\text{-}NH_4^+$	Lit - Sar	20.8	23.7	0.878	0.3798
	30°C		Rhy - Sar	42.6	20.5	2.073	0.1145
			Lit - Rhy	40.01	23.2	1.728	0.1662
		$N\text{-}NO_3^-$	Lit - Sar	36.92	23.2	1.595	0.1662
			Rhy - Sar	-3.09	23.2	-0.134	0.8937

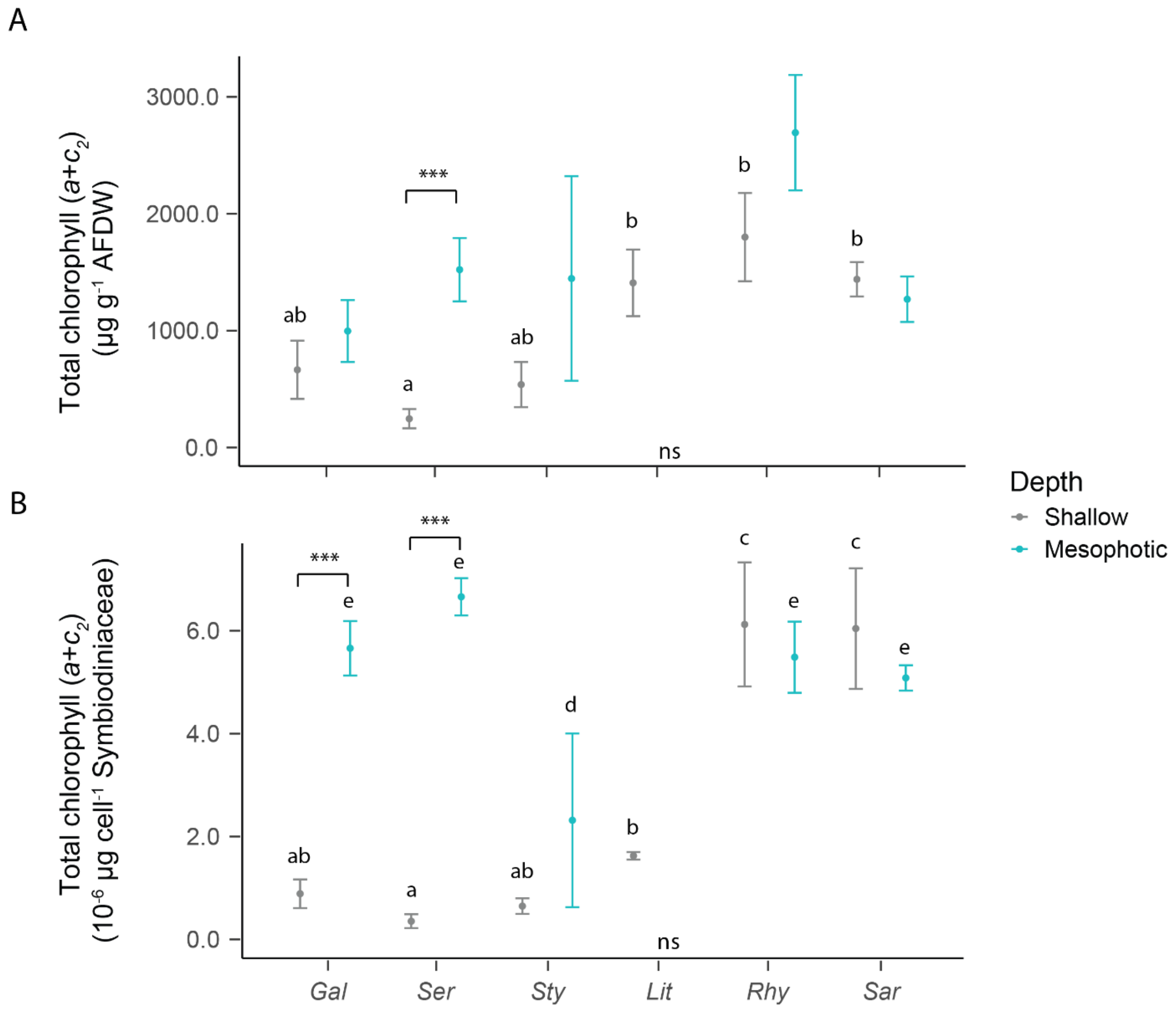

Figure S1. Total chlorophyll concentration in the species investigated. (A) Normalized to biomass (AFDW = ash-free dry weight). (B) Normalized to Symbiodiniaceae cell. The first three species (*Galaxea fascicularis*, *Seriatopora hystrix*, *Stylophora pistillata*) are hard corals and the last three (*Litophyton arboreum*, *Rhytisma fulvum fulvum*, *Sarcophyton* sp.) are soft corals. Significant differences between depths are displayed with asterisks (*** for $p < 0.001$). Significant differences between species are distinguished with letters by depth. ns = not sampled.

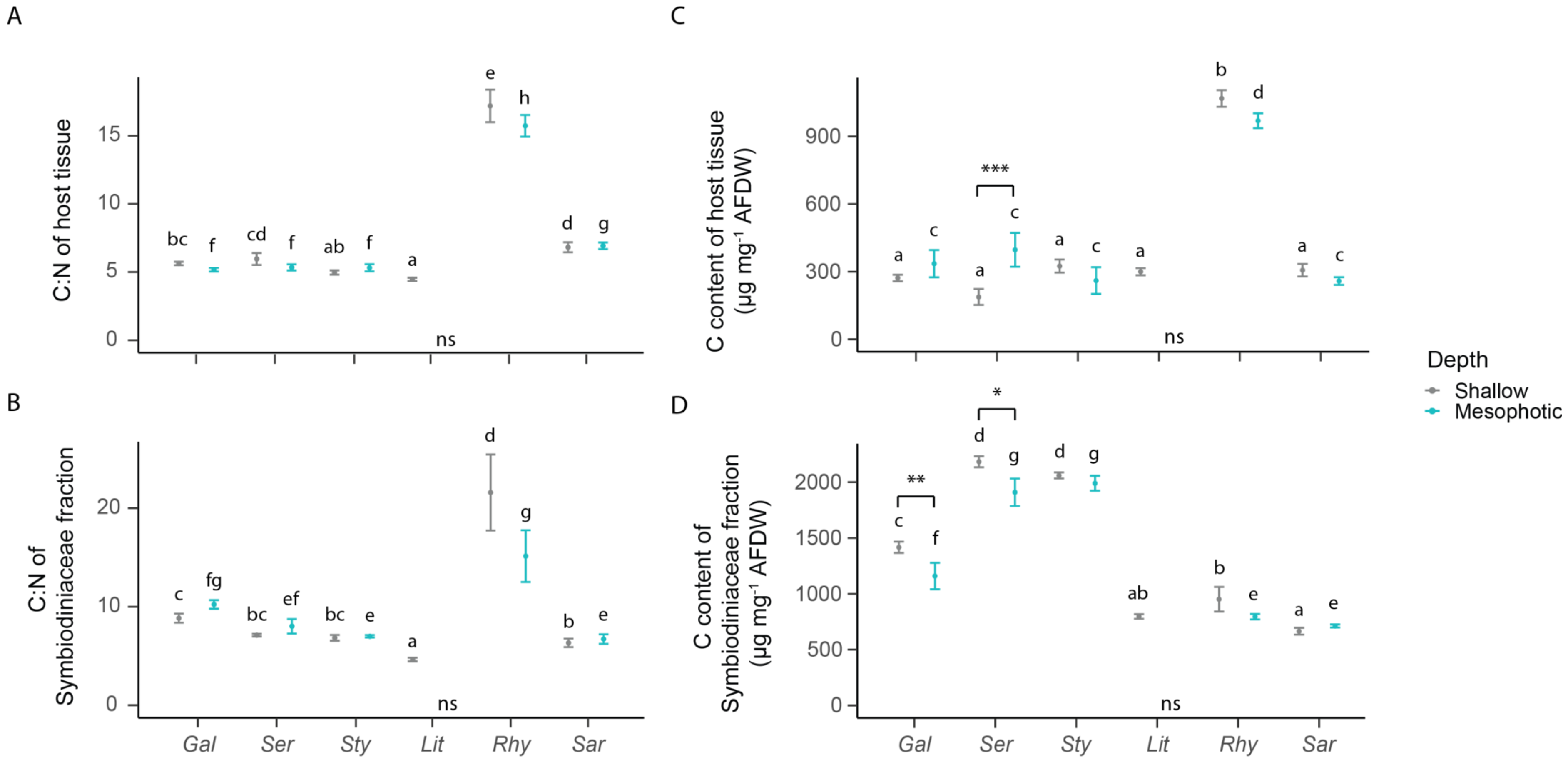

Figure S2. Elemental composition of the host tissue and Symbiodiniaceae fraction in the species investigated. (A) C:N of the host tissue. (B) C:N of the Symbiodiniaceae fraction. (C) C content of the host tissue. (D) C content of the Symbiodiniaceae fraction. The first three species (*Galaxea fascicularis (Gal)*, *Seriatopora hystrix (Ser)*, *Stylophora pistillata (Sty)*) are hard corals and the last three (*Litophyton arboreum (Lit)*, *Rhytisma fulvum fulvum (Rhy)*, *Sarcophyton* sp. (*Sar*)) are soft corals. Significant differences between depths are displayed with asterisks (* for $p < 0.05$; ** for $p < 0.01$; *** for $p < 0.001$) and significant differences between species are distinguished with letters by depth. AFDW = ash-free dry weight. ns = not sampled.

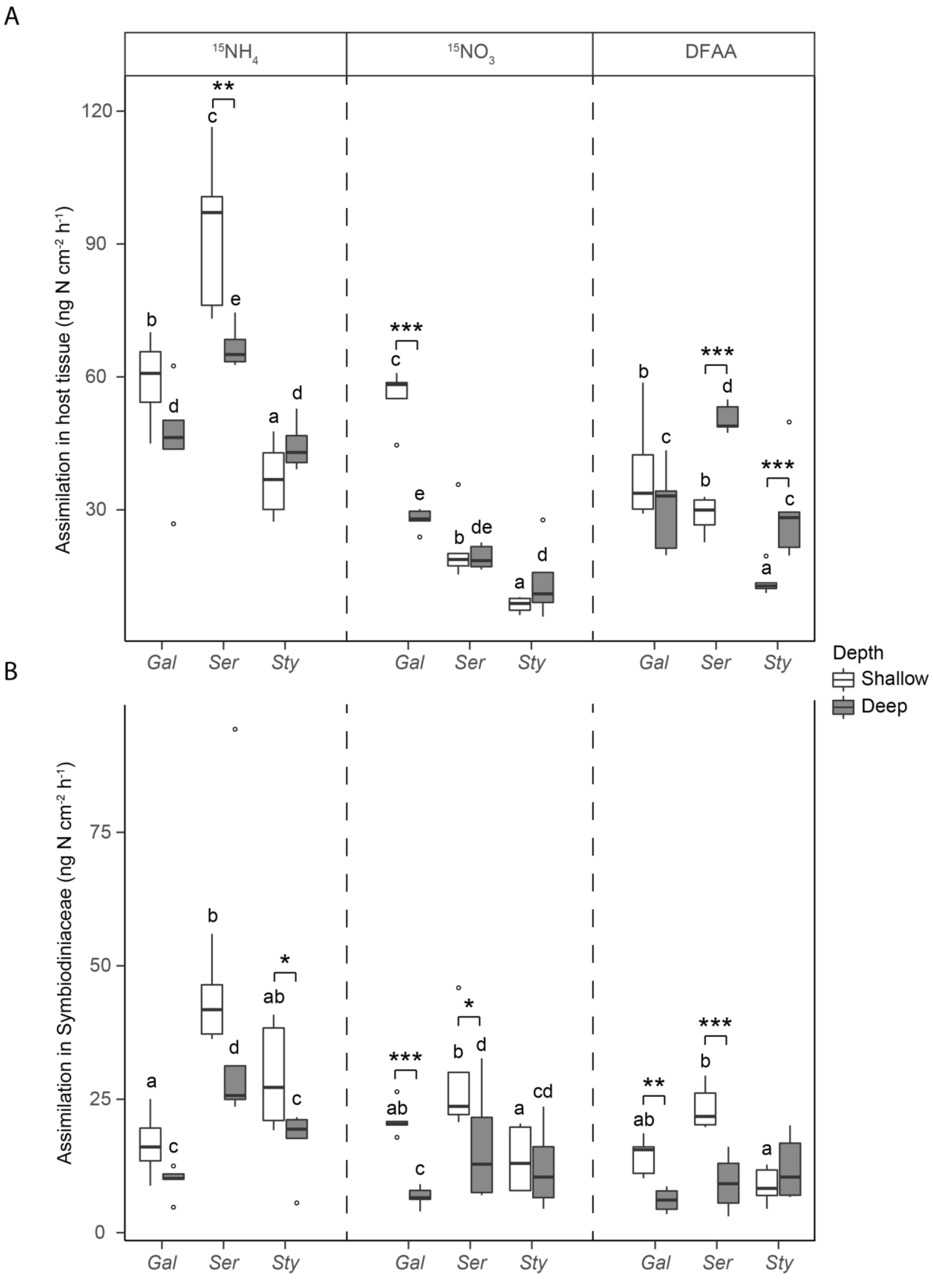

Figure S3. Assimilation rates of dissolved nitrogen in hard corals, normalized to surface area of the skeleton, in (A) the host tissue and (B) the Symbiodiniaceae fraction. Significant differences between depths are displayed with asterisks (* for $p < 0.05$; ** for $p < 0.01$; *** for $p < 0.001$). Significant differences between species are distinguished with letters by depth. Gal = *Galaxea fascicularis*. Ser = *Seriatopora hystrix*. Sty = *Stylophora pistillata*.
